# Platelet spleen tyrosine kinase is a key regulator of anti-PF4 antibody–induced immunothrombosis^∗^^†^

**DOI:** 10.1182/bloodadvances.2024014167

**Published:** 2024-12-24

**Authors:** Jan Zlamal, Vera M. Ripoll, Christine S.M. Lee, Filip Toma, Karina Althaus, Flavianna Rigoni, Andreas Witzemann, Shane Whittaker, David Capraro, Günalp Uzun, Tamam Bakchoul, Vivien M. Chen

**Affiliations:** 1 Institute for Clinical and Experimental Transfusion Medicine, Medical Faculty of Tuebingen, University Hospital of Tuebingen, Tuebingen, Germany; 2 Centre for Clinical Transfusion Medicine, Tübingen, Germany; 3 ANZAC Research Institute, Sydney Local Health District, Sydney, New South Wales, Australia; 4 Department of Haematology, Concord Repatriation General Hospital and New South Wales Health Pathology, Sydney, New South Wales, Australia; 5 Concord Clinical School, The University of Sydney, Sydney, New South Wales, Australia; 6 Division of Genome Science and Cancer, John Curtin School of Medical Research, The Australian National University, Canberra, ACT, Australia

## Abstract

Vaccine-induced immune thrombotic thrombocytopenia (VITT) is a rare but serious prothrombotic adverse event after vaccination with adenovector-based COVID-19 vaccines. Laboratory findings indicate that anti–platelet factor 4 (PF4) immunoglobulin G antibodies are the causing factor for the onset of thromboembolic events in VITT. However, molecular mechanisms of cellular interactions, signaling pathways and involvement of different cell types in VITT antibody–mediated thrombosis are not fully understood. Moreover, uncertainty exists regarding current treatment protocols because the sole anticoagulation was shown to be inefficient to prevent thrombosis progression in severe VITT cases. In this study, we demonstrate that platelet spleen tyrosine kinase (SYK) modulates anti-PF4 VITT-mediated thrombus formation in an ex vivo model of immunothrombosis. Our study showed that the selective inhibition of SYK can abrogate VITT antibody–driven procoagulant platelet formation, activation of plasmatic coagulation as well as platelet-leukocyte interplay. Most importantly, the specific inhibition of SYK in platelets but not in neutrophils prevented VITT antibody–induced multicellular thrombus formation, without perturbing the platelet function. Our findings indicate that the specific targeting of platelet SYK might be a promising therapeutic approach to prevent thrombotic complications in patients with antibody-mediated immunothrombosis.

## Key Points

• SYK pathway is critical for intercellular interaction induced by anti-PF4 antibodies in VITT.
• Inhibition of platelet SYK prevents thrombus formation without affecting platelet function.

## Visual Abstract

**Figure:**
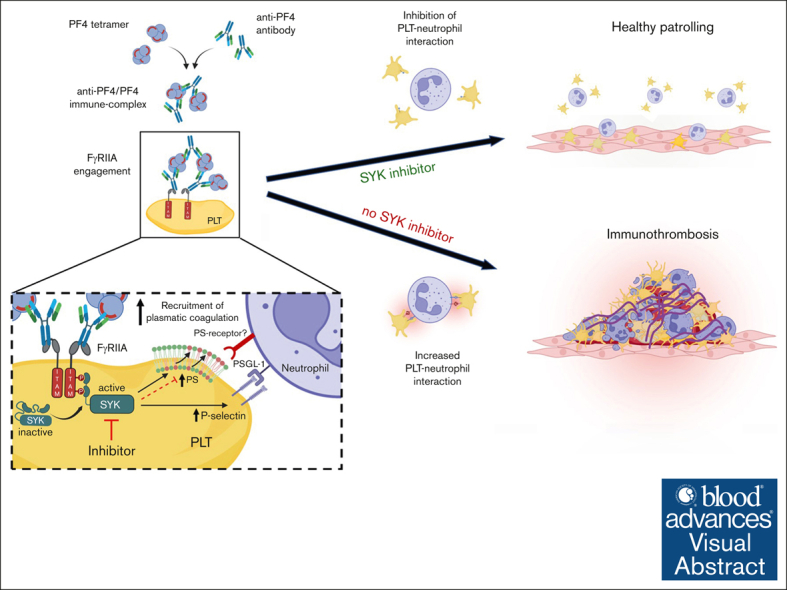

## Introduction

Facing the world-wide COVID-19 pandemic in 2020, the rapid development of adenovector-based vaccines was an essential step in the global fight against the severe acute respiraratory syndrome coronavirus type 2.^1^ However, soon after the introduction of these vaccines, increased reports of a rare but severe life-threatening adverse event, later termed as vaccine-induced immune thrombotic thrombocytopenia (VITT), raised uncertainties regarding the use of such vaccines and resulted in a prompt withdrawal of adenovector-based vaccines in many jurisdictions.^2^, ^3^, ^4^

VITT is a prothrombotic disorder caused by antibodies (Abs) from the immunoglobulin G (IgG) subclass that target the highly positive charged endogenous chemokine platelet (PLT) factor 4 (PF4; CXCL4), similar to heparin-induced thrombocytopenia (HIT).^5^^,^^6^ However, a major difference between VITT and HIT Abs is that the former seems to be independent of heparin and target highly positively charged amino acid sequences in the heparin-binding site of PF4.^7^ Recent studies identified that these VITT anti-PF4 Abs have the potential to activate PLTs and neutrophils via FcγRIIA mediated pathways, however, the mechanistic pathways leading to VITT-mediated multicellular immunothrombosis are not fully characterized.^8^, ^9^, ^10^

This study was conducted in 2 reference laboratories for HIT/VITT diagnosis and research at Tübingen and Sydney, respectively, to explore the intercellular and molecular mechanism underlying thrombosis in VITT. We found that anti-PF4 VITT Abs induce a procoagulant PLT phenotype that drives PLT-leukocyte interactions and thrombus formation. Furthermore, the specific spleen tyrosine kinase (SYK) inhibition in PLTs prevented VITT-driven procoagulant PLT formation significantly reducing the PLT-leukocyte interplay and thrombus formation.

## Methods

### Patient cohorts

Blood samples from patients with VITT referred to our laboratories were used in this study. Patient samples were withdrawn at the time of hospital admission before antithrombotic therapy or treatment with IV immunoglobulin (IVIG) was initiated. For further details, see supplemental Methods.

### Ab-mediated formation of procoagulant PLTs

Flow cytometry (FC)–based protocols were used in this study to investigate the formation of procoagulant PLTs using washed PLTs (Tübingen) and whole-blood (WB) samples (Sydney), as previously described.^11^, ^12^, ^13^, ^14^ For further details, see supplemental Methods.

### Analysis of VITT Ab–induced mechanomolecular signaling mechanisms

To investigate the pathway of PLT activation by VITT Abs, washed PLTs or WB were pretreated with the FcRIIA blocking monoclonal Ab (mAb) IV.3 (STEMCELL Technologies, Vancouver, Canada), before the addition of control or VITT Ab/plasma.

To investigate the contribution of PLT SYK to Ab-induced PLT alterations, WB samples or PLTs were pretreated with different SYK inhibitors including R406, the active metabolite of the clinically used SYK inhibitor fostamatinib, PRT-060318 (PRT-318), which was reported to inhibit thrombus formation in an in vivo model of HIT as well as the selective second- and next-generation inhibitors entospletinib (GS-9973) and lanraplenib, respectively, (GS-9876; all from Selleck, Houston, TX) for 30 minutes at room temperature (RT) before the incubation with VITT IgGs (Tübingen) or VITT positive plasmas (Sydney), respectively.^15^ All SYK inhibitors used in this study were used at concentrations that were sufficient to prevent procoagulant PLT formation induced by crosslinking of monoclonal anti–FcγRIIA AT10 with goat anti-mouse F(ab’)_2_ (both from Thermo Fisher Scientific, Waltham, MA [data not shown]).

### Thrombin generation

VITT Ab–induced thrombin generation (TG) on platelet-rich plasma (PRP) was detected using Calibrated Automated Thrombogram (Stago, Maastricht, The Netherlands), as previously described.^14^^,^^16^ For further details, see supplemental Methods.

### PLT-leukocyte interaction

#### FC

Citrated WB from healthy volunteers (50 μL) was incubated with either R406, lanraplenib, or vehicle control (dimethyl sulfoxide) for 15 minutes at RT before stimulation with 5 μM thrombin receptor-activating peptide (SFLLRN; Auspep, Sydney, Australia) and VITT or control plasma, as previously described.^11^^,^^12^ Reactions were stopped by 20-fold dilution with Hanks’ balanced salt solution (Carl Roth, Karlsruhe, Germany), which was followed by staining with Abs against CD45-BUV395 (HI30), CD41-BV510 (HIP8; BD Biosciences, NJ), CD15-FITC (C3D1; Dako, Santa Clara, CA), CD14-PerCp5.5 (MϕP9; BD Pharmingen, San Diego, CA), CD62P-PE (Psel.KO2.3) or isotype control (both from eBioscience, San Diego, CA), and (GSAO-AF647( (4-(N-(S-glutathionylacetyl)amino)phenylarsonous acid) or GSCA-AF647 control compound (4-(N-(S-glutathionylacetyl)amino)benzoic acid). For further details, see supplemental Methods.

#### Confocal microscopy

The remainder of diluted samples were stained with CD41-BV510 (HIP8; BD Biosciences, NJ), CD15-FITC (C3D1; Dako, Santa Clara, CA) and GSAO-AF647, and placed into Horm collagen (Takeda, Linz, Austria) precoated chamber slides (Thermo Fisher, Waltham, MA), which was followed by imaging using confocal microscopy. For further details, see supplemental Methods.

### Investigation of Ab-induced thrombus formation

To investigate thrombus formation by VITT Abs on a cellular level, an ex vivo model (BioFlux 200; Fluxion Biosciences, Alameda, CA) was used as previously described.^16^^,^^17^ Four microfluidic-based experimental settings were designed to (1) assess the impact of SYK inhibition in WB; (2) investigate PLT dependency; (3) explore the role of leukocyte SYK; and (4) investigate the role of PLT SYK in VITT-mediated thrombus formation. For further details, see supplemental Methods.

### Statistical analysis

Statistical analysis was performed using GraphPad Prism, version 10.1.0 (GraphPad, La Jolla, CA). Data representing comparison of means are represented as mean ± standard error of the mean. Data representing comparison of individual data points are represented as mean ± standard deviation. Comparison between groups was performed by Mann-Whitney *U* test for unpaired data sets, and paired sample *t* test for paired data sets. Comparison between multiple groups was performed by 1-way analysis of variance with the Dunnett multiple comparison test. *P* values <.05 were considered statistically significant.

Studies involving human material and blood collection were approved by the ethics committee of the Medical Faculty, Eberhard-Karls University, Tübingen, Germany (236/2021BO2 and 224/2021BO2) and Sydney Local Health District human research ethics committees (X21-0405 and 2021/ETH11929). Healthy blood donors for functional assays gave written informed consent. All studies were conducted in accordance with the Declaration of Helsinki.

## Results

### Clinical characteristics of the VITT cohort

Sera/plasma from 6 and 10 patients with laboratory and clinically confirmed VITT that were referred to the university hospitals of Tübingen and Sydney, respectively, were used in this study (Table 1). All patients tested positive for anti-PF4 IgG Abs in enzyme immune assay (mean optical density, 2.91 [range, 2.07-3.48 (Tübingen)] and 2.46 [range, 1.90-2.95 (Sydney)]). The diagnosis of VITT was further confirmed by a positive functional assay result observed in 6 of 6 (100%, Tübingen) and 10 of 10 (100%, Sydney) using PF4-induced procoagulant PLT assay or PF4 modified serotonin release, respectively.^18^

**Table 1.** **Patient characteristics**

Variable	Tübingen	Sydney
No. of patients	6	10
Sex, (female)	3 (50%)	7 (70%)
Age, y	36 (20-62)	65 (34-74)
PLT count (at admission)∗	42 (14-60)	48 (18-124)
D-dimer	31 (12.4-73)	40† (19.8-132)
Mean OD EIA	2.91 (2.07-3.48)	2.46 (1.90-2.95)
Positive PF4-modified HIPA (Tübingen)/SRA (Sydney)	6 (100%)	10 (100%)
**Vaccine**		
AstraZeneca	5 (83%)	10 (100%)
Johnson & Johnson	1 (17%)	0
Days after vaccination	9 (7-28)	10 (4-25)
**Thrombosis**	6 (100%)	10 (100%)
Cerebral venous sinus thrombosis	4 (67%)	2 (20%)
Splanchnic thrombosis	0	3 (30%)
Deep vein thrombosis	0	6 (60%)
Pulmonary embolism	1 (17%)	1 (10%)
Internal jugular vein thrombosis	0	1 (10%)
Arterial thrombosis	2 (33%)	0

Continuous variables are expressed as median (range).

EIA, enzyme immune-assay; HIPA, heparin-induced platelet activation assay; OD, optical density; SRA, serotonin-release assay.

∗ Reference range PLT count (150 × 10^9^ to 450 × 10^9^/L).

† Upper limit of normal.

### VITT Abs induce procoagulant PLTs and increase TG via SYK-dependent signaling pathway

**Figure 1.**
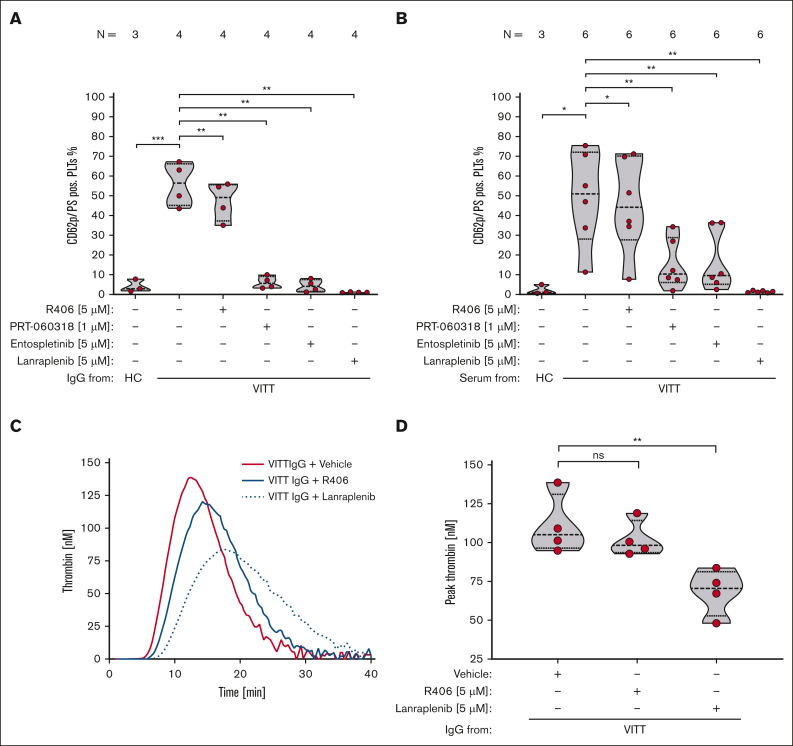
**VITT Abs induce procoagulant PLTs and increased TG via SYK-dependent signaling pathways.** (A-B) PLTs were incubated with isolated IgG (A) or (B) serum from patients with VITT or HCs in the presence of exogenous PF4 (10 μg/mL) and tested for changes in the expression levels of P-selectin (CD62p) and PS with FC. As indicated, PLTs were preincubated with SYK inhibitors R406 (5 μM), PRT-060318 (1 μM), entospletinib (5 μM), and lanraplenib (5 μM), respectively. The number of patients tested is reported in each graph. Unpaired or paired *t* test is shown. (C) Representative TG curve induced on PLTs after incubation with IgGs from 1 patient with VITT in the presence of vehicle (red line), R406 (5 μM, blue solid line) or lanraplenib (5 μM, dashed blue line). Each curve represents the amounts of generated thrombin over time. (D) Data were quantified as peak thrombin generated (nM) using Thrombinoscope software and GraphPad Prism, version 10.1.0. Violin plots showing the distribution of the values were generated using GraphPad Prism, version 10.1.0. Paired *t* test is shown. ∗*P* < .05; ∗∗*P* < .01; ∗∗∗*P* < .001. N, number of patients tested; ns, nonsignificant; PS, phosphatidylserine.

FC analysis and TG assay showed that VITT Abs induce a procoagulant PLT phenotype that harbors the capability to activate plasmatic coagulation (Figure 1A-B; supplemental Figure 1).

Next, we aimed to identify potential therapeutic targets that might prevent VITT Ab–induced procoagulant PLT formation and subsequent prothrombotic effects. Although specific blockade of PLT FcγRIIA with mAb IV.3, a mAb that specifically inhibits FcγRIIA signaling in PLTs, resulted in a near complete abolishment of VITT Ab–induced procoagulant PLT formation in vitro (*P* = .002; supplemental Figure 2), this approach has not entered clinical evaluation due to reported side effects in animal models.^19^^,^^20^ Therefore, we sought to inhibit SYK, which is a key signaling regulatory enzyme downstream of FcγRIIA.^21^^,^^22^

VITT IgG-induced procoagulant PLT formation was abolished by selective SYK inhibitors PRT-060318 (*P* = .002), entospletinib (*P* = .002), and lanraplenib (*P* = .001; Figure 1A). Of note, R406 (*P* = .005) showed moderate, however not biological relevant reduction of VITT IgG-induced procoagulant PLT formation (Figure 1A).

A significant reduction of procoagulant PLT formation was also observed upon incubation of PLTs with patient sera indicating that the SYK inhibitors are also effective in the complex proinflammatory milieu of VITT serum (Figure 1B). Most importantly, compared with vehicle and R406, significant less thrombin was generated on the PLT surface when PLTs were pretreated with lanraplenib before VITT Ab (*P* = .004; Figure 1C-D).

**Figure 2.**
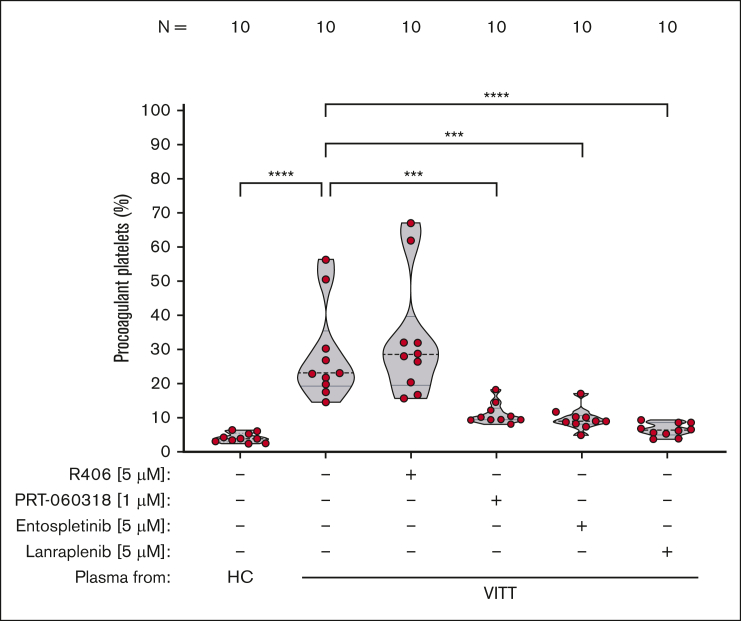
**SYK inhibition significantly reduces VITT plasma–induced procoagulant PLT response in WB.** WB from healthy individuals was pretreated with SYK inhibitors R406 (5 μM), PRT-060318 (1 μM), entospletinib (5 μM), and lanraplenib (5 μM), or vehicle control (dimethyl sulfoxide [DMSO]) for 15 minutes before exposure to PLT agonist SFLLRN (5 μM) and HC or VITT plasma. Procoagulant PLTs were enumerated by FC. One-way analysis of variance with the Dunnett's multiple comparison test was performed. The number of patients tested is reported in each graph. ∗∗∗*P* < .001;∗∗∗∗*P* < .0001. N, number of patients tested.

Next, we sought to investigate whether SYK inhibitors have the potential to prevent the intercellular interaction induced by VITT Abs. VITT plasmas induced significant increase of procoagulant PLTs in WB (*P* < .0001; Figure 2), which was abrogated by the pretreatment of WB with IV.3 (*P* < .0001; supplemental Figure 3). Most importantly, a significant reduction was observed when WB was pretreated with selective SYK inhibitors: PRT-060318 (*P* < .0001), entospletinib (*P* < .0001), as well as lanraplenib (*P* < .0001; Figure 2; supplemental Figure 4B-D; supplemental Table 1). This shows efficacious inhibition of VITT plasma-mediated procoagulant PLT formation in a concentration-dependent manner by pharmacological targeting of SYK.

Surprisingly, the clinically approved R406 failed to inhibit VITT plasma–induced procoagulant PLT formation in WB, even at concentrations of up to 10 μM (Figure 2; supplemental Figure 4A; supplemental Table 1). Notably, and in line with the findings obtained in experiments performed using isolated PLTs, the presence of SYK inhibitors did not affect the expression of the surface PLT activation marker CD62p (supplemental Figures 5 and 6).

### Inhibition of SYK prevents thrombus formation by VITT Abs

**Figure 3.**
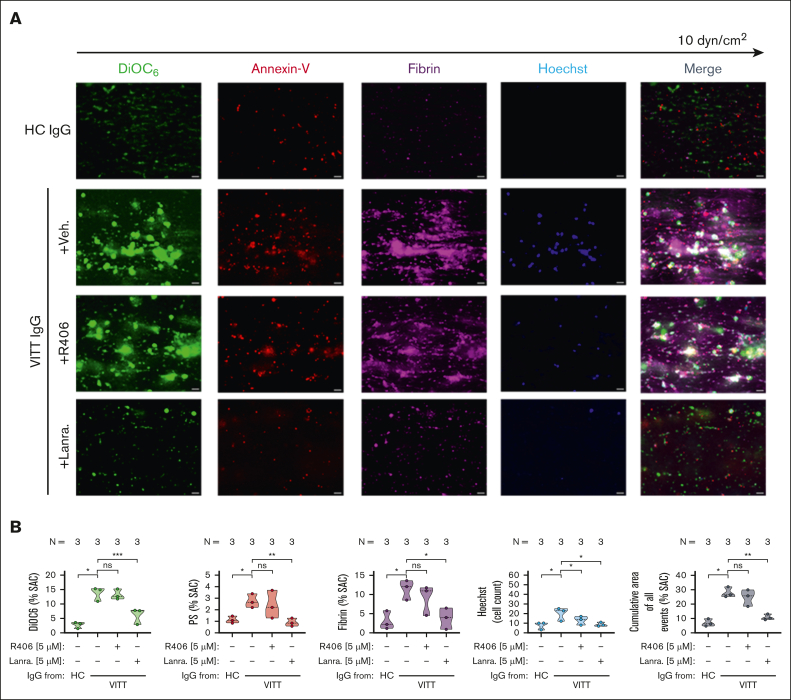
**WB SYK inhibition prevents from VITT Ab–mediated multicellular thrombus formation ex vivo.** (A-B) Whole blood from healthy individuals was incubated with IgG from HCs or patients with VITT in the presence of exogenous PF4 (10 μg/mL) and SYK inhibitors R406 or lanraplenib (both 5 μM) or vehicle control before recalcification and perfusion through microfluidic channels at a venous shear rate of 250 sec^–1^ (10 dyne/cm^2^) for 25 minutes. (A) After perfusion, images were acquired at magnification ×40. Scale bar, 20 μm. (B) Violin plots showing the percentage of total surface area coverage (% SAC) by DiOC_6_ (nonprocoagulant PLTs), annexin-V (procoagulant PS), fibrin, count of Hoechst-positive–labeled cells (leukocytes); and cumulative total % SAC with DiOC_6_, PS, and fibrin–labeled thrombus captured in the microfluidic channel. The number of patients tested is reported in each graph. Unpaired or paired *t* test is shown. ∗*P* < .05; ∗∗*P* < .01; ∗∗∗*P* < .001. N, number of patients tested; ns, nonsignificant.

To verify the inhibitory effect of SYK on VITT-associated thrombosis, a microfluidic assay was used. VITT IgGs mediated increased multicellular thrombus formation ex vivo (*P* < .05; Figure 3). Compared to healthy control (HC), VITT IgGs mediated increased deposition of nonprocoagulant PLTs (mean % surface area coverage DiOC_6_ [3,3’-dihexyloxacarbocyanine iodide] ± standard error of the mean: *P* < .05; Figure 3), procoagulant PLTs (*P* < .05; Figure 3) as well as increased recruitment of leukocytes to the thrombus area (*P* < .05; Figure 3). Moreover, VITT but not HC IgGs were detected to activate the plasmatic coagulation cascade, as a significant increase in fibrin network generation was observed in WB samples that were spiked with VITT IgG (*P* < .05; Figure 3).

Pretreatment of WB with lanraplenib before coincubation with VITT IgG resulted in a significant inhibition of VITT IgG-mediated multicellular thrombus formation in the microfluidic model of Ab-mediated thrombosis (*P* = .002; Figure 3). Compared with vehicle, the presence of lanraplenib resulted in a significant reduction in the deposition of non-procoagulant (*P* = .0006; Figure 3) and in a near complete abolishment of procoagulant PLT deposition (*P* = .009; Figure 3). Additionally, lanraplenib also prevented VITT Ab–mediated activation of the plasmatic coagulation cascade and attraction of leukocytes to thrombus, as less fibrin (*P* = .011; Figure 3) and reduced numbers of leukocytes (*P* = .041; Figure 3) were detected after perfusion of lanraplenib pretreated WB samples, respectively. Interestingly, although a decrease in leukocyte recruitment was also observed in WB samples that were pretreated with R406 (*P* = .022; Figure 3), the presence of R406 did not result in a reduction in the total area of VITT Ab–mediated thrombus (Figure 3). Compared with vehicle, no alterations in the deposition of nonprocoagulant (Figure 3), procoagulant PLTs (Figure 3), as well as in fibrin network generation (Figure 3) were observed in R406-pretreated and VITT IgG-incubated WB samples upon perfusion through the microfluidic system.

### VITT Abs drive increased PLT-leukocyte cross talk in a SYK-dependent manner

**Figure 4.**
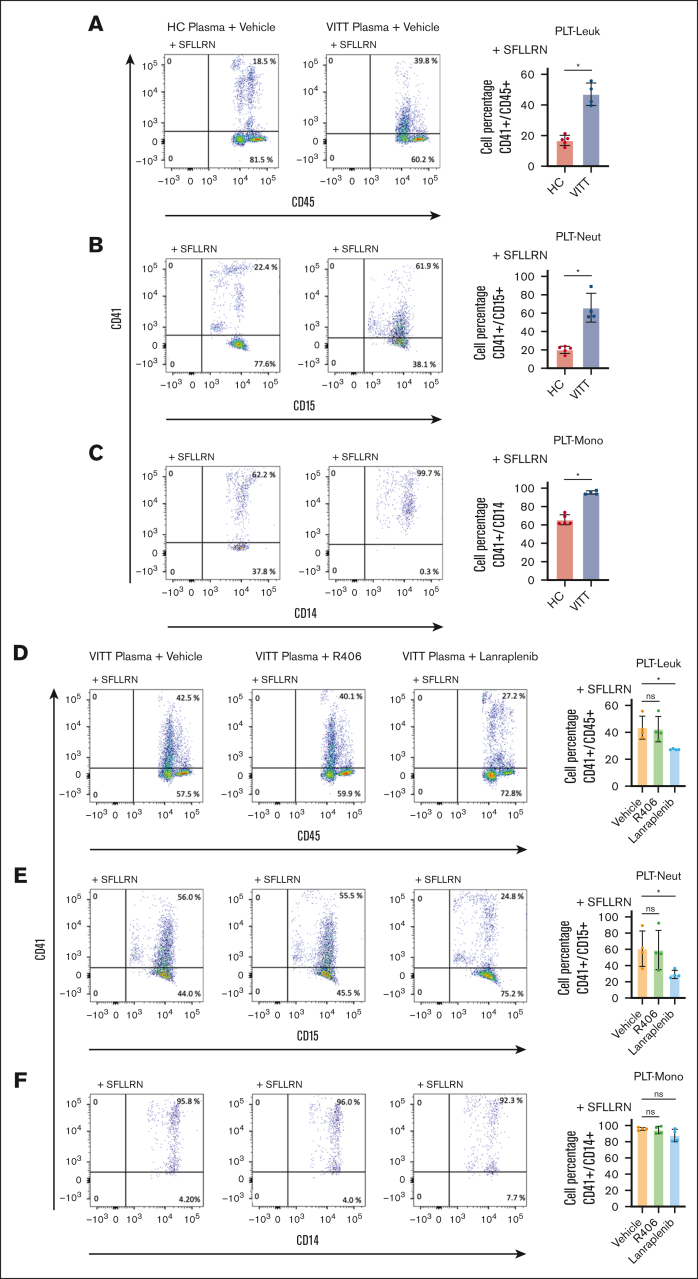
**VITT plasma induces increased PLT-leukocyte (Leuk) interaction via SYK-dependent pathways.** Representative FC dot plots and quantification of (A) PLT-Leuk (CD41/CD45 double positive), (B) PLT-neutrophil (Neut; CD41/CD15 double positive), and (C) PLT-monocyte (Mono; CD41/CD14 double-positive events) interaction in WB after incubation with SFLLRN (5 μM) and VITT (n = 4) or HC plasma (n = 5). Impact of SYK inhibitor pretreatment on VITT plasma–induced (D) PLT-Leuk (CD41/CD45 double positive), (E) PLT-Neut (CD41/CD15 double positive), and (F) PLT-Mono interactions (CD41/CD14 double positive) in WB. As indicated, WB was pretreated with SYK inhibitor R406 or lanraplenib (5 μM), respectively. Aggregate data in bar graph showing the distribution of the values from n = 4 individual plasma samples. Unpaired or paired *t* test is shown. ∗*P* < .05. ns, nonsignificant.

Having observed the reduction of leukocyte recruitment to thrombus in the presence of SYK inhibitors in WB, we assessed the effect of SYK on VITT-mediated PLT-leukocyte interplay. PLT-leukocyte aggregates were quantified by FC in WB samples in the presence of protease-activated receptor-1 agonist, SFLLRN, and plasma from HCs or VITT patients. SFLLRN (5 μM) resulted in >95% CD62p expression (supplemental Figure 6) and elevated PLT-leukocyte aggregates compared with buffer (supplemental Figure 7). Addition of VITT plasma resulted in a significant increase of PLT-leukocyte aggregate formation compared with HC plasma (*P* = .016; Figure 4A). Both PLT-neutrophil (*P* = .016; Figure 4B) as well as PLT-monocyte aggregates (*P* = .016; Figure 4C) contributed to the increase of PLT-leukocyte aggregates observed in the presence of plasma from patients with VITT.

SYK inhibition with lanraplenib but not R406 prevented VITT Ab–induced increased PLT-leukocyte aggregate formation (*P* = .999 and *P* = .040; Figure 4D). Interestingly, the reduction of PLT-leukocyte aggregates was mostly due to lanraplenib-mediated inhibition of PLT-neutrophil aggregates (*P* = .040; Figure 4E) whereas PLT-monocyte aggregates were minimally affected (Figure 4F).

### SYK pathway is essential for the PLT-leukocyte cross talk in VITT

**Figure 5.**
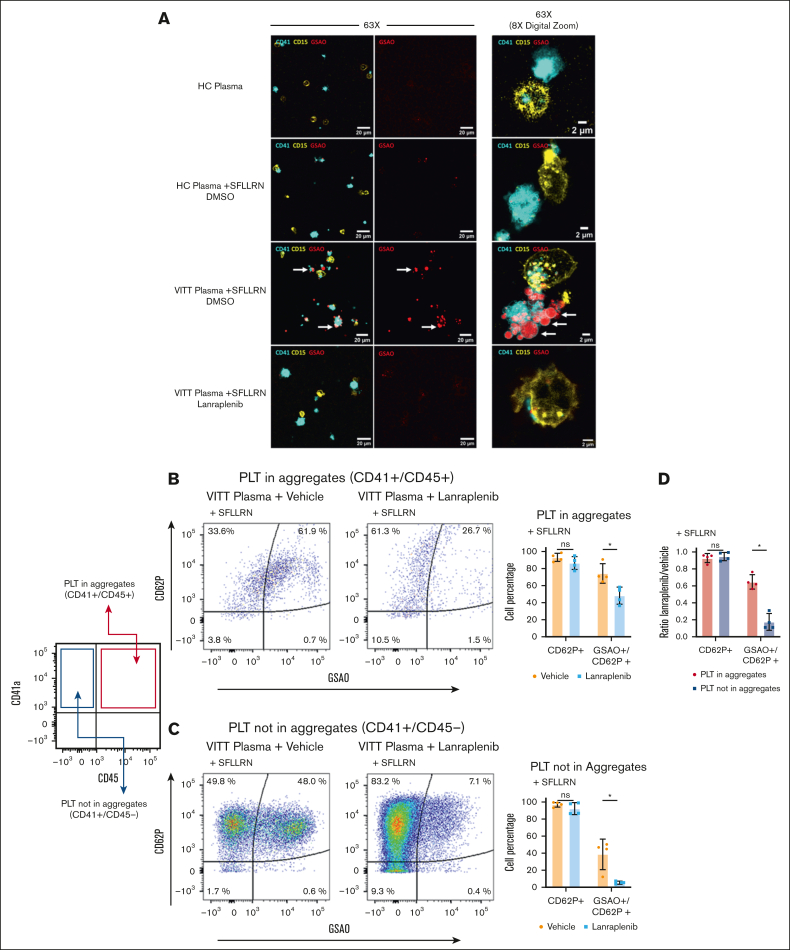
**VITT Ab–mediated PLT-Leuk interactions are predominantly driven by the procoagulant platelet subpopulation.** (A) Representative confocal images of WB from healthy individuals costained with anti-CD41 (PLTs, cyan), anti-CD15 (Neuts, yellow), and GSAO-AF647 (procoagulant PLTs, red) after incubation with SFLLRN (5 μM) and plasma from patients with VITT or HCs. As indicated, WB samples were pretreated with SYK inhibitor lanraplenib (5 μM) or vehicle control before incubation with SFLLRN and VITT plasma. Images were acquired at magnification ×63 with additional ×1 or ×8 digital zoom (n = 4). Scale bars, 20 and 2 μM, respectively. Representative images of independent experiments are shown. White arrows highlight GSAO-AF647–positive (red) ballooned procoagulant PLTs interacting with Neuts (yellow). (B-C) Dot plots and quantification of FC analysis from corresponding WB samples from panel [A]. CD62p (P-selectin)–positive PLTs were compared with (CD62p/GSAO double positive) procoagulant PLT events in the presence of lanraplenib (5 μM) or vehicle in (B) PLT-Leuk aggregates (CD41/CD45 double positive, [PLTs in aggregates]) and (C) single PLTs (CD41^+^/CD45^−^ events, [PLTs not in aggregates]). (D) Effect of lanraplenib pretreatment (expressed as ratio of PLT phenotype lanraplenib to control) on P-selectin expression and procoagulant PLT subpopulation within the PLT-Leuk aggregates (CD41/CD45 double positive) and PLTs not in aggregates (CD41^+^/CD45^−^ events) was compared. Note that CD62p–positive events are the combination of CD62p single-positive and CD62p/GSAO double-positive events. Combined data reported in bar graphs showing the distribution of the values from n = 4 individual plasma samples. Unpaired or paired *t* test is shown ∗*P* < .05. ns, nonsignificant.

Because VITT Abs were detected to cause increased formation of procoagulant PLTs as well as PLT-leukocyte interaction, we investigated the contribution of procoagulant PLTs to increased PLT-leukocyte interplay. Confocal microscopy analysis enabled visualization of procoagulant PLTs (GSAO positive) as well as PLT-leukocyte aggregates. Both increased GSAO-positive procoagulant PLTs and increased PLT-leukocyte aggregates were observed in samples incubated with VITT plasma compared with HCs (Figure 5A). Digital magnification demonstrated incorporation of procoagulant PLTs in the PLT-neutrophil aggregates (Figure 5A). Importantly, pretreatment with lanraplenib prevented VITT plasma–induced procoagulant PLT formation, reduction of PLT-leukocyte aggregates, and reduction of procoagulant PLTs in the remaining PLT-neutrophil aggregates (Figure 5A).

Quantitation of this interaction by FC further revealed that pretreatment with lanraplenib of VITT plasma and SFLLRN-treated WB did not affect the proportion of PLTs expressing CD62p within PLT-leukocyte aggregates (*P* = .200; Figure 5B). In contrast, lanraplenib significantly reduced the proportion of VITT Ab–induced procoagulant PLTs within CD41/CD45 double-positive aggregates (*P* = .004; Figure 5B; supplemental Figure 8A-B). Analysis of the CD45^−^ PLT events within the same samples suggested that lanraplenib was more efficient at reducing procoagulant PLT responses of single PLTs (*P* = .003; Figure 5C; supplemental Figure 8C-D). Comparison of the effect of lanraplenib in CD45^+^ PLT events compared with the CD45^−^ PLT events demonstrated no change in CD62p with a significantly lower reduction of procoagulant PLTs in CD45^+^ PLT aggregates compared with CD45^−^ PLT events (lanraplenib-to-vehicle ratio ± standard deviation: *P* < .029; Figure 5D), suggesting PLTs within PLT-leukocytes aggregates are more likely to remain procoagulant, even in the presence of SYK inhibition.

### PLT SYK has a central role in VITT Ab–mediated thrombus formation

Leukocyte activation and subsequent neutrophil extracellular trap (NET)–derived prothrombotic mediators have been reported to cause PLT activation and subsequent coagulopathy.^23^^,^^24^ As SYK inhibitors are not cell specific, we aimed to dissect the contribution of PLT SYK in VITT Ab–mediated thrombus formation. When PLTs were depleted from WB samples before incubation with VITT IgGs, no significant thrombus formation was observed in our model, even when unstimulated PLTs were added back to the samples that were incubated with VITT Abs (supplemental Figure 9A-B). Of note, no increased recruitment of neutrophils could be observed when PLT-depleted WB was incubated with VITT IgG before reconstitution with unstimulated PLTs (*P* = .0011; supplemental Figure 9A-B). Moreover, no changes in VITT Ab–mediated thrombus formation were observed when PLT-depleted WB containing leukocytes and red blood cells were pretreated with SYK inhibitor R406 or lanraplenib before reconstitution with VITT Ab–induced procoagulant PLTs (supplemental Figure 10).

**Figure 6.**
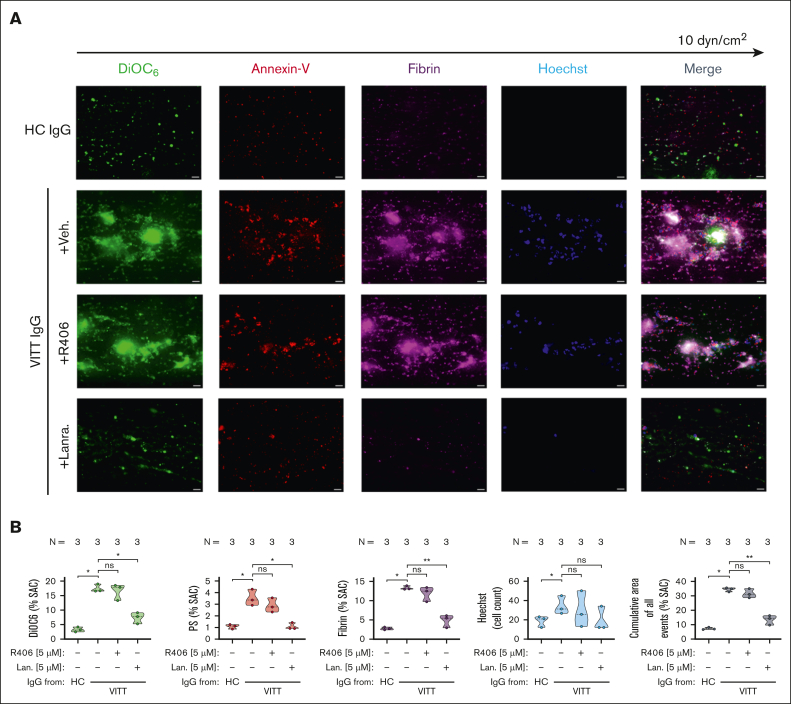
**VITT Ab–induced procoagulant PLT-mediated thrombus formation can be prevented via the inhibition of SYK in PLTs.** (A-B) PLTs from healthy individuals were incubated with IgG from patients with VITT in the presence of exogenous PF4 (10 μg/mL) and indicated SYK inhibitors or vehicle control (upper panel) before reconstitution into autologous PLT-depleted WB and perfusion through microfluidic channels at a venous shear rate of 250 sec^–1^ (10 dyne) for 25 minutes. (A) After perfusion, images were acquired at magnification ×40. Scale bar, 20 μm. (B) Violin plots showing the percentage of total (% SAC) by DiOC_6_, PS, fibrin(ogen), count of Hoechst-positive–labeled cells; and cumulative total % SAC of DiOC_6_, PS, and fibrin(ogen)-labeled thrombus captured in the microfluidic channel. Unpaired or paired *t* test is shown. ∗*P* < .05; ∗∗*P* < .01; ∗∗∗*P* < .001. N, number of patients tested; ns, nonsignificant.

Finally, we investigated the specific effect of SYK inhibition in PLTs. Pretreatment of isolated PLTs with the selective SYK inhibitor lanraplenib but not R406 before VITT Ab incubation resulted in a significant reduction of VITT Ab–mediated multicellular thrombus formation ex vivo (*P* = .008; Figure 6). At a concentration that did not affect PLT aggregatory function (supplemental Figure 11), the presence of lanraplenib led to a significant reduction of procoagulant PLTs (*P* = .018) integration into the growing thrombus (Figure 6). Moreover, compared with vehicle and R406, lanraplenib also inhibited procoagulant PLT-mediated activation of plasmatic coagulation as a significant decrease in fibrin generation was detected in the microfluidic system (*P* = .005; Figure 6). Interestingly, pretreatment of PLTs with lanraplenib as well as R406 resulted in only minor reduction in leukocyte adhesion in the observation window (Figure 6).

## Discussion

Our study demonstrates that VITT Abs induce procoagulant PLT-neutrophil interplay via stimulation of PLT–Fc-RIIA–associated SYK leading to multicellular thrombus formation and activation of the plasmatic coagulation system. Most importantly, our data showed that the next-generation selective SYK inhibitor lanraplenib can prevent VITT Ab–induced PLT-leukocyte interaction and thrombus formation, without impairing the hemostatic response of PLTs. Collectively, our findings suggest that specific targeting of PLT SYK might be a promising therapeutic approach to treat thrombocytopenia and thrombosis in VITT, and possibly in other Fc-RIIA–mediated immune thrombotic diseases.

VITT is associated with severe thromboembolic complications. An important clinical observation that was obtained from the first VITT cases suggested that anticoagulation alone might be insufficient to prevent progression of thrombosis induced by the anti-PF4 Abs.^25^ Interestingly, high-dose IV immunoglobulin was shown to be effective in reducing PLT activation by VITT Abs, probably by interfering with the Fc-RIIA signaling.^26^, ^27^, ^28^ This observation motivated us to dissect the molecular mechanisms of PLT activation as well as cell-cell interaction in VITT, with a special focus on the potential therapeutic use of inhibitors of the FcγRIIA-SYK axis in PLTs.

In our study, VITT Abs were found to induce a subpopulation of procoagulant PLTs in a Fc-RIIA–dependent manner, which results in an increased activation of plasmatic coagulation (as observed by TG on the procoagulant PLT surface) as well as upregulation of PLT-leukocyte interaction (as shown in WB FC). Those events finally led to the formation of a multicellular thrombus with increased fibrin deposition in our ex vivo model of immunothrombosis. Since our data indicated that VITT Ab–induced procoagulant PLT formation could be prevented by an Fc-RIIA–blocking moAb, we thought to investigate the inhibition of SYK, a key regulatory nonreceptor tyrosine kinase downstream of Fc-receptors.^29^^,^^30^ The formation of procoagulant PLTs by VITT Abs was investigated in a FC-based screening approach, testing clinically approved R406, as well as selective SYK inhibitors PRT-060318, entospletinib, and lanraplenib. Our screening studies showed acceptable reduction in the procoagulant PLT subpopulation for all tested agents other than R406 in washed PLTs as well as WB samples, respectively. Because PRT-060318 and entospletinib are not approved for clinical use and PRT-060318 showed an impairment of PLTs aggregatory function, we performed more comprehensive experiments using fostamatinib (R406), a clinically approved drug to decrease PLT clearance in immune thrombocytopenia by downregulating Fc-γ receptor–mediated phagocytosis, and the novel, more selective SYK inhibitor lanraplenib (GS-9876), which is currently trialed for acute myeloid leukemia (ClinicalTrials.gov identifier: NCT05028751).^31^, ^32^, ^33^, ^34^ Expanding on the results of Smith et al, potent SYK inhibitor, lanraplenib, but not R406, showed high efficacy in preventing VITT Ab–induced procoagulant PLT formation as well as increased TG on the PLT surface.^35^

Interestingly, while some previous studies demonstrated R406 could mitigate collagen receptor glycoprotein VI (GPVI)-or HIT Ab FcγRIIA–mediated PLT aggregation,^36^^,^^37^ in our hands, SYK inhibitors, other than PRT-060318, at the same concentrations used in our procoagulant PLT studies, were unable to inhibit PLT aggregation in response to high-dose collagen in PRP. This may be partially explained, by differences between washed PLTs and PRP aggregation studies, however, Spalton et al. also found that, although R406 inhibited phosphorylation of SYK, phospholipase Cγ2 and linker for activation of T cells with low concentrations of GPVI agonists collagen, collagen-related peptide, and convulxin, partial recovery was observed in response to higher concentrations, most likely because of incomplete blockade of SYK.^35^^,^^37^^,^^38^ Our study is, to our knowledge, the first that has focused on the procoagulant PLT phenotype and we clearly show, across 2 laboratories, that R406 was not able to prevent VITT Ab–mediated procoagulant PLT-driven thrombus formation. As R406 lacked efficacy to prevent from VITT Ab–induced FcγRIIA–mediated procoagulant effects in our experiments, we speculate that VITT IgG Ab FcγRIIA–mediated stimulus acts in a similar manner to high concentrations of GPVI agonists in the context of R406-mediated SYK inhibition. Furthermore, the newer SYK inhibitors have more selectivity and lower dissociation constants (Kd) (entospletinib Kd = 7.6 nM) compared with R406 (Kd = 15-100 nM), which may account for the observed differences between inhibitors.

Additionally, differences in the signaling pathways between VITT and HIT may be contributing. VITT Abs were reported to specifically target the heparin-binding site within PF4, whereas HIT Abs are directed to the polar regions of PF4.^5^^,^^7^ Recent findings highlighted an important role of the thrombopoietin receptor cellular myeloproliferative leukemia protein in PF4-mediated PLT activation.^39^ Conformational changes of PF4 by heparin-independent anti-PF4 Abs could serve as an additional amplification of signaling that can only be abrogated by selective SYK inhibitors.

Another important finding from our study is the SYK dependency of the dominantly observed interaction between procoagulant PLTs and neutrophil granulocytes in VITT. Recent studies showed that neutrophil activation and subsequent NET formation contribute to endothelial dysfunction and a prothrombotic environment in VITT.^23^ In fact, mechanistic studies revealed that VITT Abs induce NET formation via engagement of Fc-RIIA expressed on neutrophils leading to increased NET-mediated PLT activation and formation of PLT-neutrophil–rich thrombi ex vivo as well as in vivo.^24^ Although several groups suggested that the presence of PLTs is an important requirement for these effects, the specific PLT subpopulations and potential ligand–receptor interaction partner that are involved in VITT Ab–mediated PLT-leukocyte interplay are not elucidated yet.^40^^,^^41^ Using FC, we found that procoagulant PLTs have high preference to interact with neutrophils in VITT. This finding might be explained by the elevated level of P-selectin on the surface of procoagulant PLTs, which might lead to an increased PLT-neutrophil aggregation via the P-selectin/P-selectin glycoprotein ligand-1 axis.^42^^,^^43^ However, in our experimental setting, no increased PLT-leukocyte interplay was detected in HCs, despite the presence of protease-activated receptor-1agonist SFLLRN, which increases P-selectin expression on the PLT surface. Most importantly, the reduction of phosphatidylserine (PS) externalization using SYK inhibitor lanraplenib resulted in significantly lower PLT-neutrophil aggregate formation despite the presence of VITT Abs and SFLLRN whereas formation of PLT-monocyte aggregates remained mostly unaffected. This finding directs toward a pivotal role of PLT PS for being an important mediator of the increased PLT-neutrophil interplay in VITT, similar to data reported from an in vivo model of ischemic stroke.^44^ Additionally, the observation that SYK inhibitors are not able to prevent PLT-monocyte interaction could be explained due to alternative pathways that are not prevented by short-term SYK inhibitor pretreatment of WB. We speculate that VITT Ab–mediated release of tissue factor by monocytes, as observed in HIT, may account for the differences between neutrophils and monocyte-PLT interaction.^45^ However, this interesting point should be addressed in future investigations. Taken together, our results indicate that PS is an important mediator of increased PLT-leukocyte interactions in VITT.

Multicellular interplay as well as the interaction with plasmatic factors of the coagulation system contribute to vessel occlusion in thrombotic diseases.^46^^,^^47^ To expand our FC findings, we used an ex vivo microfluidic-based model of Ab-mediated thrombosis. In this model, VITT Abs caused multicellular thrombus supporting our hypothesis that Ab-induced procoagulant PLTs are important mediators of increased PLT-leukocyte interplay as well as strong activator of the plasmatic coagulation cascade. The need for procoagulant PLTs was reinforced by the finding that no thrombus was formed by the direct incubation of VITT Abs with PLT-depleted WB samples from healthy donors, even when native PLTs (not exposed to VITT Abs) were added back to blood samples before perfusion. Of note, the pretreatment of leukocytes with SYK inhibitors before reconstitution with VITT Ab–induced procoagulant PLTs did not mitigate VITT Ab–mediated thrombus emphasizing the importance of PLT SYK in VITT Ab–mediated thrombus formation.

Although our data provide further insights regarding the role of PLT SYK as critical mediator of the VITT-mediated prothrombotic coagulopathy, some technical aspects might limit immediate translation. First, quantitative differences in VITT Ab–mediated procoagulant PLTs were observed in FC assays performed in both centers, though the qualitative findings were the same. This variation could be most likely explained because of well-known variations in VITT Ab potency from patients with VITT, differences in donor Fc-RIIA responsiveness as well as differences in FC platforms (isolated PLTs vs. WB) used. Additionally, the use of a collagen-coated surface in our microfluidic system might overemphasize the role of PLTs in VITT Ab–mediated immunothrombosis. However, because PLTs were detected for being essential components in thrombi that were retrieved from patients with severe VITT, we believe that our studies highlight the role of PLTs in the complex pathophysiology of VITT.^23^^,^^48^ This hypothesis is further supported because conventional anti-PLT drugs as well as anticoagulants showed limited efficacy in the treatment of VITT.

Taken together, our data indicate that lanraplenib might be a suitable candidate for treatment of Ab-induced Fc-RIIA–mediated thrombotic diseases. This new therapeutic concept of using SYK inhibitors to treat Ab-mediated immunothrombosis provides an important clinical advantage besides preventing activation of the coagulation system and inflammatory response. Many patients with VITT presented with cerebral sinus venous thrombosis.^49^^,^^50^ The anticoagulation of these patients harbors the risk of congestive bleeding, especially when thrombocytopenia coexists. Of note, SYK inhibition was shown to have a good safety profile in patients with thrombocytopenia with no evidence for increased bleeding risk in treated patients with immune thrombocytopenia.^32^^,^^51^^,^^52^ Of a great clinical interest is the finding that VITT Ab–mediated procoagulant PLTs and increased thrombin were inhibited in our study at lanraplenib concentrations, that did not perturb PLTs activation response and aggregatory function, which may indicate a safe pharmacological profile of lanraplenib in patients who are thrombocytopenic. However, although our study might provide new insights into the pathophysiology of VITT, the safety and efficacy of lanraplenib in preventing Ab-induced immunothrombosis need to be further investigated in in vivo approaches and subsequent clinical trials.

Our study provides another piece of knowledge of the complex pathophysiology of VITT. Given the increasing recognition of anti-PF4–mediated thrombocytopenic thrombotic conditions without proximate exposure to heparin and vaccine, SYK inhibition alone or together with anticoagulation might be a potential therapeutic approach to treat anti-PF4 Ab–mediated immunothrombosis.

Conflict-of-interest disclosure: J.Z., K.A., and T.B. submitted a patent (PCT/E P2022/06214) for the detection of procoagulant platelets as a diagnostic tool for heparin-induced thrombocytopenia and vaccine-induced thrombotic thrombocytopenia. The remaining authors declare no competing financial interests.

## Supplementary Material

Supplemental Methods, Figures, Table, and References
