# Supplementary material for: Platelet spleen tyrosine kinase is a key regulator of anti-PF4 antibody–induced immunothrombosis
Source: Blood Adv. 2024 Dec 24;9(8):1772–85. doi: 10.1182/bloodadvances.2024014167 (PMC12008526; doi:10.1182/bloodadvances.2024014167)
Supplement: Supplemental Methods, Figures, Table, and References [file BLOODA_ADV-2024-014167-mmc1.pdf]

# Supplementary Material

## Supplementary Methods

### Patient cohorts

Blood samples from patients with VITT referred to our laboratories were used in this study. Patient samples were withdrawn at the time of hospital admission before intensified antithrombotic therapy or treatment with intravenous immunoglobulin was initiated. Laboratory and clinical data were reviewed by three (Tübingen, Germany) and five (Sydney, Australia) physician experts in the field of thrombosis and haemostasis. The diagnosis of VITT was confirmed when patients presented with thrombosis with thrombocytopenia, raised D-Dimer 4-28 days after exposure to adenovirus-based COVID-19 vaccine and activating IgG anti-PF4 Abs were detected by PF4 immunoassay as well as functional PLT activating assay: modified heparin-induced platelet activation assay (HIPA) with PF4 (Germany), PF4-serotonin release assay (Australia) and procoagulant flow cytometry assay (both labs).[1] Additionally, in Tübingen, control sera were obtained from healthy individuals with blood group AB at the red cross blood donation centre. All control sera used in this study were obtained from healthy donors post COVID-19 vaccination. In Sydney, healthy control plasma was obtained from healthy non-pregnant volunteers post COVID-19 vaccination with no history of stroke, thromboembolism or other known medical conditions. Volunteers were not using antiplatelet therapy, anticoagulation or nonsteroidal anti-inflammatory drugs within 7 days.

### Antibody-mediated formation of procoagulant PLTs

Two flow cytometry-based protocols were used in this study to investigate the formation of procoagulant PLTs using washed PLTs (Tübingen) and whole blood (WB) samples (Sydney) as previously described.[2-5] Briefly, fresh whole blood (WB) from healthy donors was withdrawn by cubital venipuncture into acidic-citrate-dextrose (ACD) containing vacutainers (Becton-Dickinson, Plymouth, United-Kingdom) and allowed to rest for 45 min at 37°C. After a centrifugation step (120g, 20 min, room temperature [RT], no brake), PLT-rich-plasma (PRP) was gently separated and supplemented with apyrase (5 µL/mL; Sigma-Aldrich, St. Louis, USA) and pre-warmed ACD (333 µL/mL; Sigma-Aldrich, St. Louis, USA). After an additional centrifugation step (650g, 7 min, RT, no brake), the PLT pellet was resuspended in 5 mL of wash-solution (modified Tyrode buffer: 5 mL bicarbonate buffer, 20 percent [%] bovine serum albumin, 10% glucose solution [Braun, Melsungen, Germany], 2.5 U/mL apyrase, 1 U/µL hirudin [Pentapharm, Basel, Swiss], pH 6.3) and allowed to rest for 15 min at 37°C. Following final centrifugation (650g, 7 min, RT, no brake) wPLTs were resuspended in 2 mL of resuspension-buffer (50 mL of modified Tyrode buffer, 0.5 mL of 1 mM MgCl2, 1 mL of 2 mM CaCl2, pH 7.2) and adjusted to 300x103 PLTs/µL after the measurement at a hematological analyzer (CELL-DYN Ruby, Abott, Wiesbaden, Germany) was performed. IgG fractions from HC or VITT patients were isolated using a commercially available IgG-purification-kit (Melon™-Gel IgG Spin Purification Kit, Thermo Fisher Scientific, Waltham, USA) as previously described (mean concentration of total IgG in mg/mL: 15.6±1.5 vs. 16.7±3.3, p value 0.857).[5] wPLTs (~11x$10^6$) were then incubated with 5 µL VITT patient sera or isolated VITT IgGs for 60 min under rotating conditions at RT. After incubation, 5 µL (~1x$10^6$ wPLTs) were transferred into a final volume of 100 µL of Hank´s balanced salt solution (HBSS, Carl-Roth, Karlsruhe Germany, [137 mM NaCl, 1.67 mM $CaCl_2$, 5.5 mM glucose]) and 1 µL anti-CD62p-

APC (BD, San Jose, USA) and 1 µL Annexin-V-FITC (Immunotools, Friesoythe, Germany) were added for 30 min at RT in the dark. Procoagulant PLTs were identified as PLTs that express CD62p and Annexin-V positive events. wPLTs that were treated with thrombin receptor activating peptide-6 (TRAP-6; [Sigma-Aldrich, St. Louis, USA]) and Convulxin (0.1 µg/mL, [Enzo Life Sciences, Lörrach, Germany]) served as positive controls. Afterwards, PLTs were resuspended with HBSS to a final volume of 500 µL and immediately assessed via flow cytometry (FC; Navios, Beckman-Coulter, Brea, USA).

In Sydney, WB (13 µL) was incubated with 5 µM SFLLRN peptide (Auspep, Parkville, Australia) and 5 µL citrated VITT patient plasma for 10 min in a 50 µL total reaction volume containing 2.5 mM Gly-Pro-Arg-Pro peptide (Sigma Aldrich, St. Louis, USA) and 2.5 mM calcium chloride in HBSS. The reaction was stopped by diluting the 50 µL reaction mix with 150 µL HBSS, followed by staining with antibodies to CD45-AF488 (HI30, [Stemcell Technologies, Vancouver, Canada]), CD41-BV510 (HIP8 [BD Biosciences, New Jersey, USA ]), P-selectin-PE (Psel.KO2.3), or isotype control (both from eBioscience, San Diego, USA), and GSAO-AF647 [4-(N-(S-glutathionylacetyl)amino)phenylarsonous acid] or control compound GSCA-AF647 [4-(N-(S-glutathionylacetyl)amino)benzoic acid]. Samples were fixed with PAMFix (Platelet Solutions Ltd, Nottingham, UK), centrifuged, and resuspended, before analysis on a BD LSRFortessa X-20 cytometer with acquisition of 7000 PLT events. All steps were performed at room temperature. Procoagulant PLTs were quantified as % of CD62p and GSAO dual-positive events.[3]

***Thrombin generation***

VITT Ab-induced thrombin generation (TG) on PRP was detected using Calibrated Automated Thrombogram (CAT; Stago, Maastricht, Netherlands) as previously described.[5,6] In brief, venous blood from healthy individuals was withdrawn into vacutainers containing sodium citrate 0.105 M (3.2%; BD, Plymouth, UK) and allowed to rest for 20 min at RT. PRP was prepared by centrifugation (20 min, 120g, no brake) and adjusted with autologous platelet poor plasma (PPP; 10 min, 2000g, RT) to a PLT count of 150x106/mL. Afterwards, PRP was treated with IgGs from HC or one VITT patient in the presence of exogenous PF4 (10 µg/mL) and incubated for 60 min at RT under rotating conditions. Following incubation, 80 µL of the cell suspension were dispensed into the well of round-bottom 96 well-microtitre plates (Fluoroskan Ascent, ThermoLabsystems, Helsinki, Finland) and supplemented with 20 µL of PRP-reagent containing recombinant tissue factor (Thrombinoscope BV, Maastricht, The Netherlands). 20 µL of fluorogenic substrate and calcium (FluCa-Kit reagent, Thrombinoscope BV, Maastricht, The Netherlands) were dispensed automatically by the device in each well. Fluorescence was acquired for 60 min with a 390-nm excitation/460-nm emission filter set, and parameters automatically calculated by dedicated software (Thrombinoscope BV, Maastricht, The Netherlands). Where indicated, PLTs were preincubated with SYK inhbitors R406 (5µM), Lanraplenib (5 µM) or vehicle (DMSO) for 30 min at RT prior to VITT Ab incubation.

## Platelet-leukocyte interaction

Flow cytometry (FC): Citrated WB from healthy volunteers (50 µL) was incubated with either R406, Lanraplenib or vehicle control (DMSO) for 15 min at RT. 30 µL aliquots were diluted with Hank's balanced salt solution (HBSS, pH 7.35) containing 2.5 mM GPRP (Sigma-Aldrich, St. Louis USA) and 2.5 mM calcium. Samples were then

stimulated with 5 µM thrombin receptor-activating peptide (SFLLRN, [Auspep, Sydney, Australia]) and 5 µL of VITT or control plasma for 10 min. Reactions were stopped by 20 fold dilution with HBSS and a 40 µL aliquot was stained with antibodies against CD45-BUV395 (HI30), CD41-BV510 (HIP8; [BD Biosciences, New Jersey, USA]), CD15-FITC (C3D1; [Dako, Santa Clara, USA]), CD14-PerCp5.5 (MϕP9; [BD Pharmingen, San Diego, USA]), CD62P-PE (Psel.KO2.3) or isotype control (both from eBioscience, San Diego, USA ), and GSAO-AF647 or GSCA-AF647 control. Samples were fixed with PAMFix (Platelet Solutions Ltd, Nottingham, UK), washed, resuspended, and stored at RT for 1 h in the dark before analysis on a BD LSRFortessa X-20 with acquisition of 7000 CD45 positive events.[4] The GSAO+/CD62P+ gates were defined for each experiment based on fluorescence thresholds set by the control compound GSCA-AF647 and IgG1-PE-isotype control using CD41/CD45 double positive events for the aggregate calculations and using the CD41 positive population for the PLTs not in aggregates. The CD41/CD45 double positive events had auto-fluorescence in the far-red channel (AF647) hence gates between these population and CD41 positive might differ.

Confocal microscopy: The remainder of diluted samples were stained with CD41-BV510 (HIP8; [BD Biosciences, New Jersey, USA]), CD15-FITC (C3D1; [Dako, Santa Clara, USA]) and GSAO-AF647 and placed into Lab-Tek 8-well chamber slides (Thermo Fisher, Waltham, USA) precoated with 50 µg/mL of Horm collagen (Takeda, Linz, Austria]). Imaging of stained PLTs was performed using a Leica TCS SP8 confocal microscope using a Leica HC PL APO 63x1.20 Water CORR CS2 lens at x1 digital and x8 digital zoom (magnification: x 63 and x 504). Images were processed and analysed using Huygens Professional deconvolution software and Fiji/ImageJ.[7]

***Investigation of antibody-induced thrombus formation***

To investigate the effect of VITT Abs on the coagulation cascade and subsequent thrombus formation, an *ex vivo* model (BioFlux 200, Fluxion Biosciences, Alameda, USA) was used as previously described.[5,6,8] Four different microfluidic-based experimental settings were designed to assess the impact of SYK in VITT:

i) SYK-inhibition in WB: To analyze the pancellular impact of SYK on VITT Ab-induced thrombosis, healthy donor WB was pre-treated with different SYK inhibitors prior to the addition of VITT Abs (1 hour, RT, radial rotation).

ii) PLT dependency of VITT-mediated thrombus: To confirm the contribution of PLTs in VITT Ab-mediated thrombus formation *ex vivo*, PLTs were removed from WB by several centrifugation steps. Briefly, PRP was gently removed after centrifugation (120g, 15 min, no brake) of WB samples from healthy donors which was followed by two additional washing steps (120g, 10 min, no brake) with NaCl 0.9% (Braun, Melsungen, Germany) to remove residual PLTs. Afterwards, PLT-depleted samples were incubated directly with VITT or control IgGs (1 hour, RT, radial rotation) prior to direct reconstitution with unstimulated autologous PLTs without additional washing steps.

iii) Leukocyte SYK: To explore the potential impact of SYK inhibitor on leukocytes, PLT-depleted WB samples were pre-treated with SYK inhibitors at indicated concentrations for 30 min at RT prior to reconstitution with VITT IgG incubated PLTs and immediate perfusion through the ex vivo microfluidc model .

iv) PLT SYK: For the investigation of the biological relevance of SYK in PLTs, PRP was isolated from WB samples of healthy donors (120g, 15 min, no brake) and pre-treated with SYK inhibitors prior to VITT Ab incubation (1 hour, RT, radial rotation) and

reconstitution into PLT-depleted autologous WB. SYK inhibition was achieved by pre-treating WB samples (i) or PLT-depleted WB (ii) or PLTs (iii) with R406 [5 µM], Lanraplenib [5 µM] or vehicle for 30 min at RT.

After incubation with VITT Abs, samples were labelled with 3,3′-Dihexyl-oxacarbocyaniniodid (DiOC6, 2.5 µM; Sigma Aldrich, Saint Louis, USA) which is internalized in PLTs with an intact mitochondrial transmembrane potential and therefore discriminates non-procoagulant from procoagulant PLTs.[9,10] Additionally, Alexa Fluor (AF) 647-Annexin A5 (1:200), AF 546 human Fibrinogen (8.5 µg/mL) and Hoechst 33342 (3 µg/mL; Thermo Scientific, Carlsbad, USA) were added to WB during reconstitution which was followed by recalcification and perfusion of samples through the microfluidic system at a venous shear rate of 250s-1 (10 dyne) for 25 min.

After perfusion, immunofluorescence and bright field images were taken from 3-5 randomly chosen microscopic fields at x40 magnification rate (Axio observer 7, [Carl Zeiss, Oberkochen, Germany]). Images were processed identically using adjusted threshold settings and exclusion of image artefacts by using Fiji image processing software.[7]

## Supplementary Figures and Tables

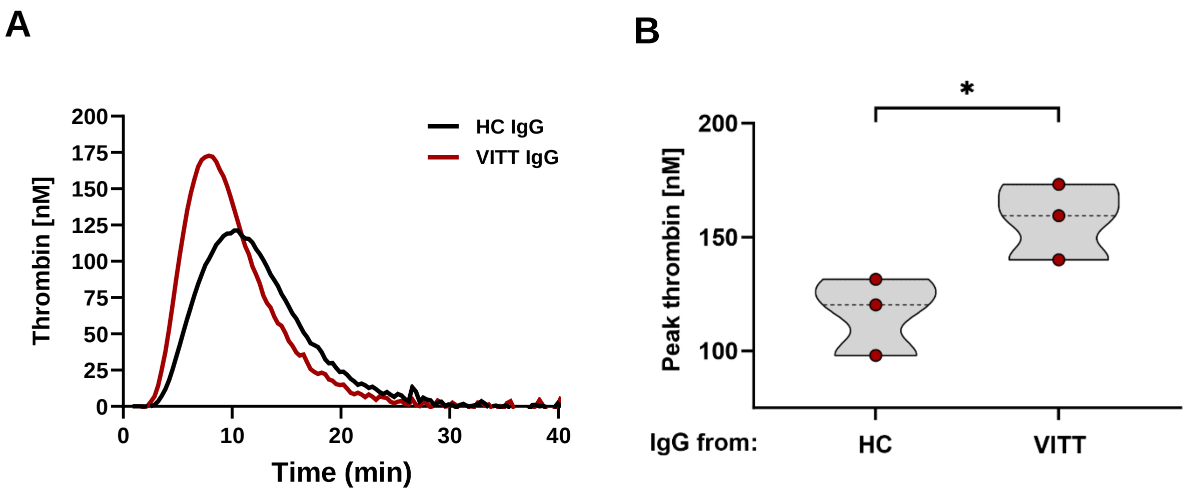

**Supplementary Figure 1-VITT antibody-induced procoagulant platelets mediate increased thrombin generation.** [**A**] Representative thrombin generation curve induced on PLTs after incubation with IgGs from HC (N=3, [black line]) or one VITT patient (red line). Each curve represents the amounts of generated thrombin over time. [**B**] Data were quantified as peak thrombin generated (nM) using Thrombinoscope software and Graphpad prism. Violin plots showing the distribution of the values were generated using Graphpad prism Version 10.1.0. The panels show results of individual experiments using at least three different donors. *P < 0.05, **P < 0.01, ***P < 0.001, and ****P < 0.0001. ns, non-significant; VITT, vaccine-induced immune thrombotic thrombocytopenia; PF4, platelet factor 4; CD62p, P-selectin; PS, phosphatidylserine. CAT, calibrated automated thrombogram; PLT, platelet; FC, flow cytometry; HC, healthy control; IgG, immunoglobulin G; CD62p, P-Selectin; PS, phosphatidylserine; N, number of samples.

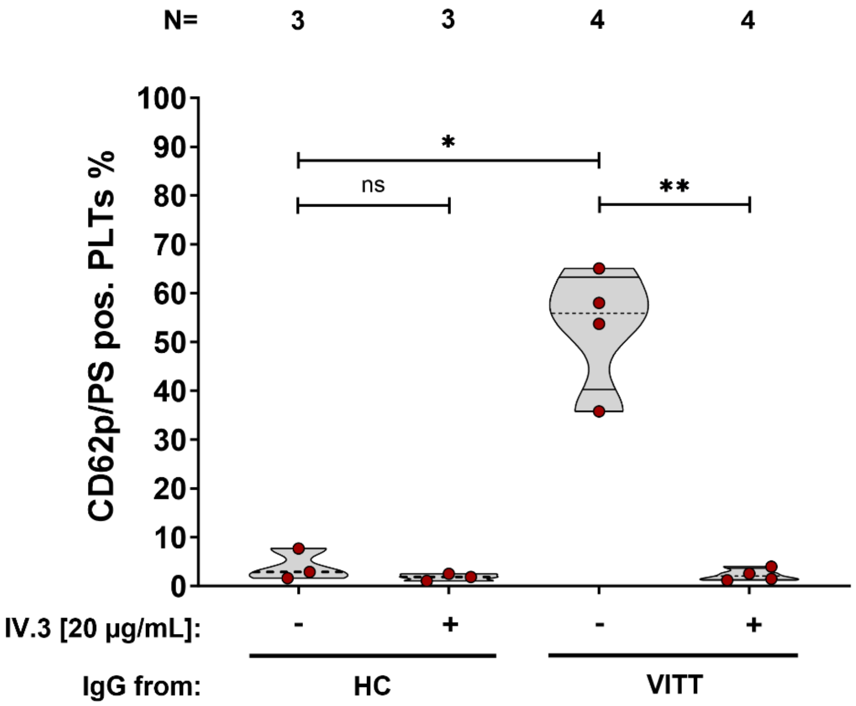


**Supplementary Figure 2-Impact of platelet Fc-gamma-RIIA blockade on VITT patient IgG-induced procoagulant platelet formation.** PLTs from healthy individuals were pre-treated with the Fc-gamma-RIIA blocking monoclonal antibody IV.3 prior to incubation with healthy control or VITT patient IgG in the presence of exogenous PF4 (10 µg/mL) and tested for procoagulant PLT formation by FC. The number of IgGs tested is reported in each graph. Violin plots showing the distribution of the values were generated using Graphpad Prism Version 10.1.0. *P<0.05, **P<0.01, ***P<0.001, and ****P< 0.0001. ns: non-significant. P values were calculated using the Mann-Whitney U test. *P<0.05, **P<0.01, ***P<0.001, and ****P< 0.0001. ns, non-significant. PF4, platelet factor 4; VITT, vaccine-induced immune thrombotic thrombocytopenia; IgG, immunoglobulin G.

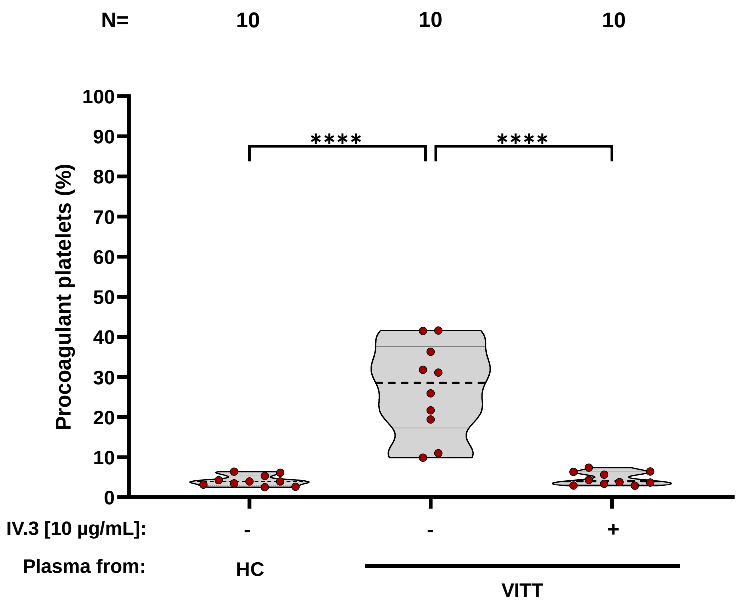


**Supplementary Figure 3-VITT patient plasma-induced formation of procoagulant platelets in whole blood is prevented by Fc-gamma-RIIA blockade.** Procoagulant platelet (PLT) response induced by VITT plasma in healthy donor whole blood was significantly reduced to levels comparable to healthy control plasma when WB was pre-treated with 10 µg/mL Fc-gamma-RIIA-blocking monoclonal antibody IV.3. The number of plasmas tested is reported in each graph. Violin plots showing the distribution of the values were generated using Graphpad Prism Version 10.1.0. One-way ANOVA with Dunnett's multiple comparisons test was performed. ****$p<0.0001$. VITT, vaccine-induced immune thrombotic thrombocytopenia.

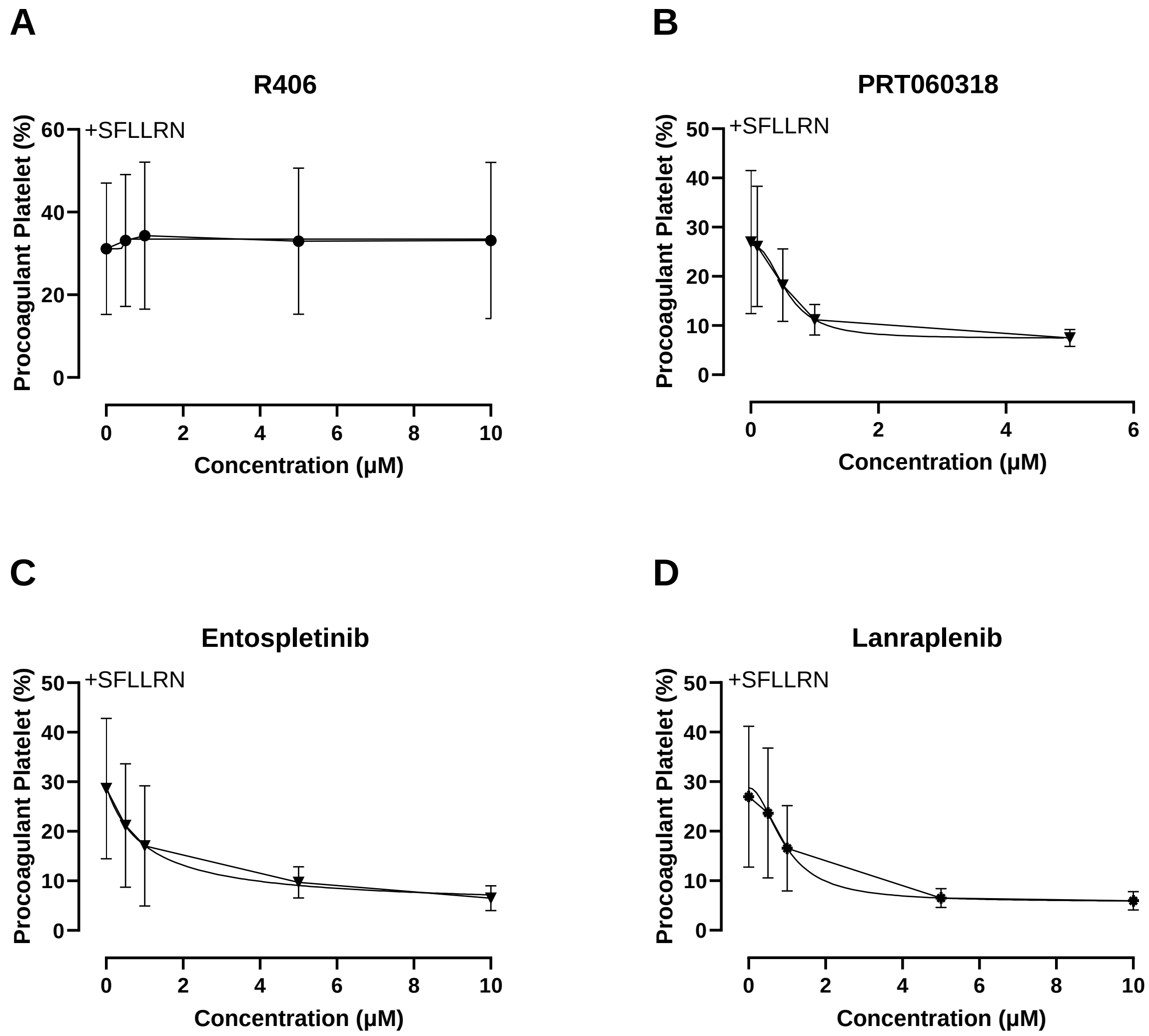


**Supplementary Figure 4-SYK inhibtors prevent VITT plasma-induced procoagulant platelet formation in a concentration-dependent manner.** Healthy donor whole blood was pretreated with SYK inhibitors (0.5-10 µM R406, 0.1-5 µM PRT-060318, 0.5-10 µM Entospletinib, 0.5-10 µM Lanraplenib or vehicle control for 15 min prior to coincubation with PAR-1 agonist SFLLRN (5 µM) and VITT plasma (N=10). Procoagulant PLT formation was assessed by FC. Error bars indicate mean ±SD. Non-linear regression with variable slope and four parameters was used to fit the datapoints for IC50 determination. FC, flow cytometry; SYK, spleen tyrosine

kinase; PAR-1, protease-activated receptor; VITT, vaccine-induced immune thrombotic thrombocytopenia.

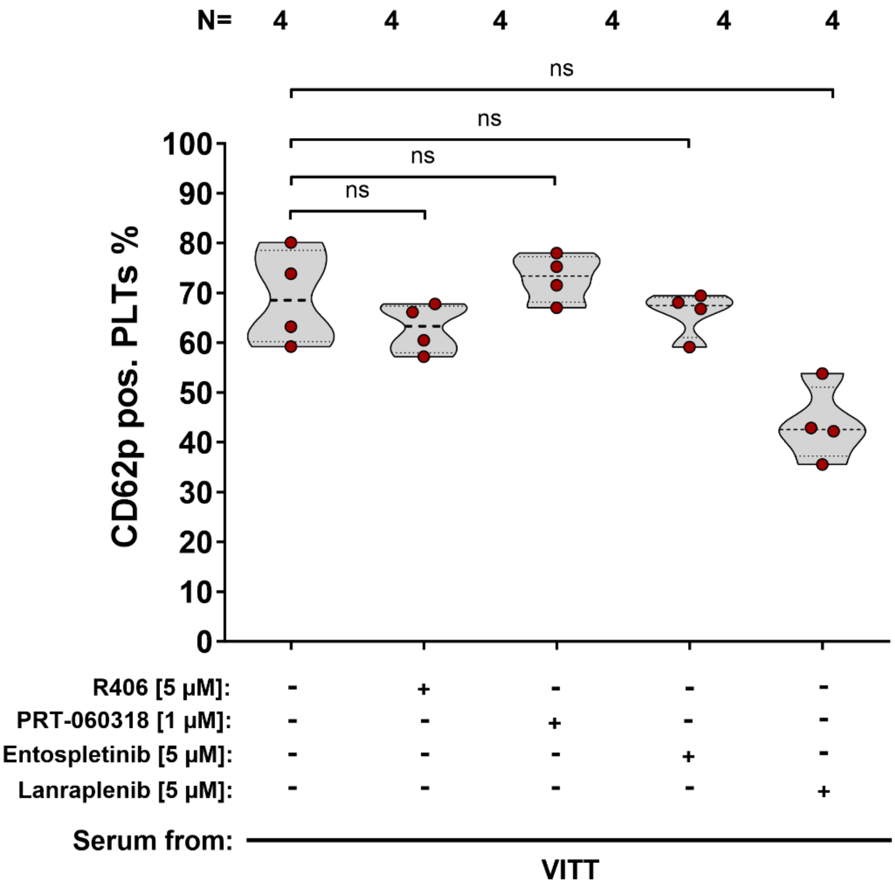


**Supplementary Figure 5-Impact of spleen tyrosine kinase inhibiton on VITT -induced P-selectin expression.** Platelets (PLTs) were incubated with sera from patients with vaccine-induced thrombotic thrombocytopenia (VITT) in the presence of exogenous PF4 (10 µg/mL) and tested for changes in the expression level of P-selectin in flow cytometry. Where indicated, PLTs were preincubated with spleen tyrosine kinase (SYK) inhibitors R406 (5 µM), PRT-318 (1 µM) and selective SYK inhibitors Entospletinib (5 µM) and Lanraplenib (5 µM), respectively. The number of sera tested is reported in each graph. Violin plots showing the distribution of the values were generated using GraphPad Prism, Version 10.1.0. ns: non-significant. *P<0.05, **P<0.01, ***P<0.001, and ****P< 0.0001. VITT, vaccine-induced immune thrombotic thrombocytopenia; PF4, platelet factor 4; CD62p, P-selectin; PS, phosphatidylserine.

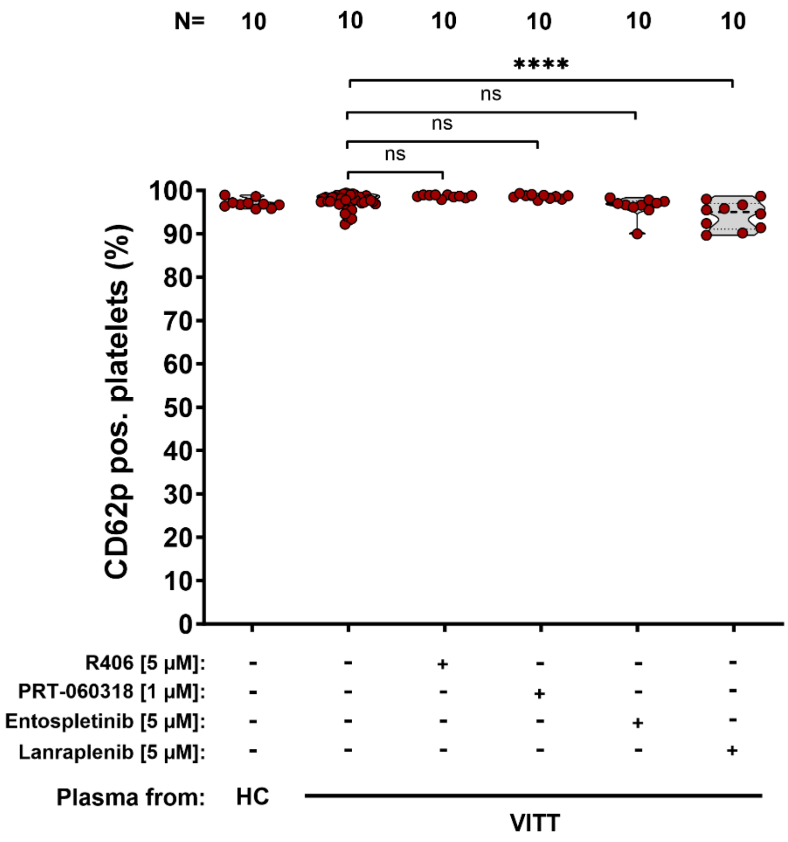


**Supplementary Figure 6-Syk inhibition results in a minimal reduction of platelet P-selectin expression in whole blood.** Whole blood from healthy individuals was pre-treated with Syk inhibitors (5 µM R406, 1 µM PRT060318, 5 µM entospletinib, 5 µM lanraplenib) or vehicle control for 15 min before exposure to platelet agonist 5 µM SFLLRN and healthy control (HC) or VITT plasma. The proportion of P-selectin (CD62p) positive platelet events was enumerated by flow cytometry. The number of plasmas tested is reported in each graph. Violin plots showing the distribution of the values were generated using GraphPad Prism, Version 10.1.0.One-way ANOVA with Dunnett's multiple comparisons test was performed. ns: non-significant. ***P<0.001 and ****P<0.0001. VITT, vaccine-induced immune thrombotic thrombocytopenia; PF4, platelet factor 4; CD62p, P-selectin.

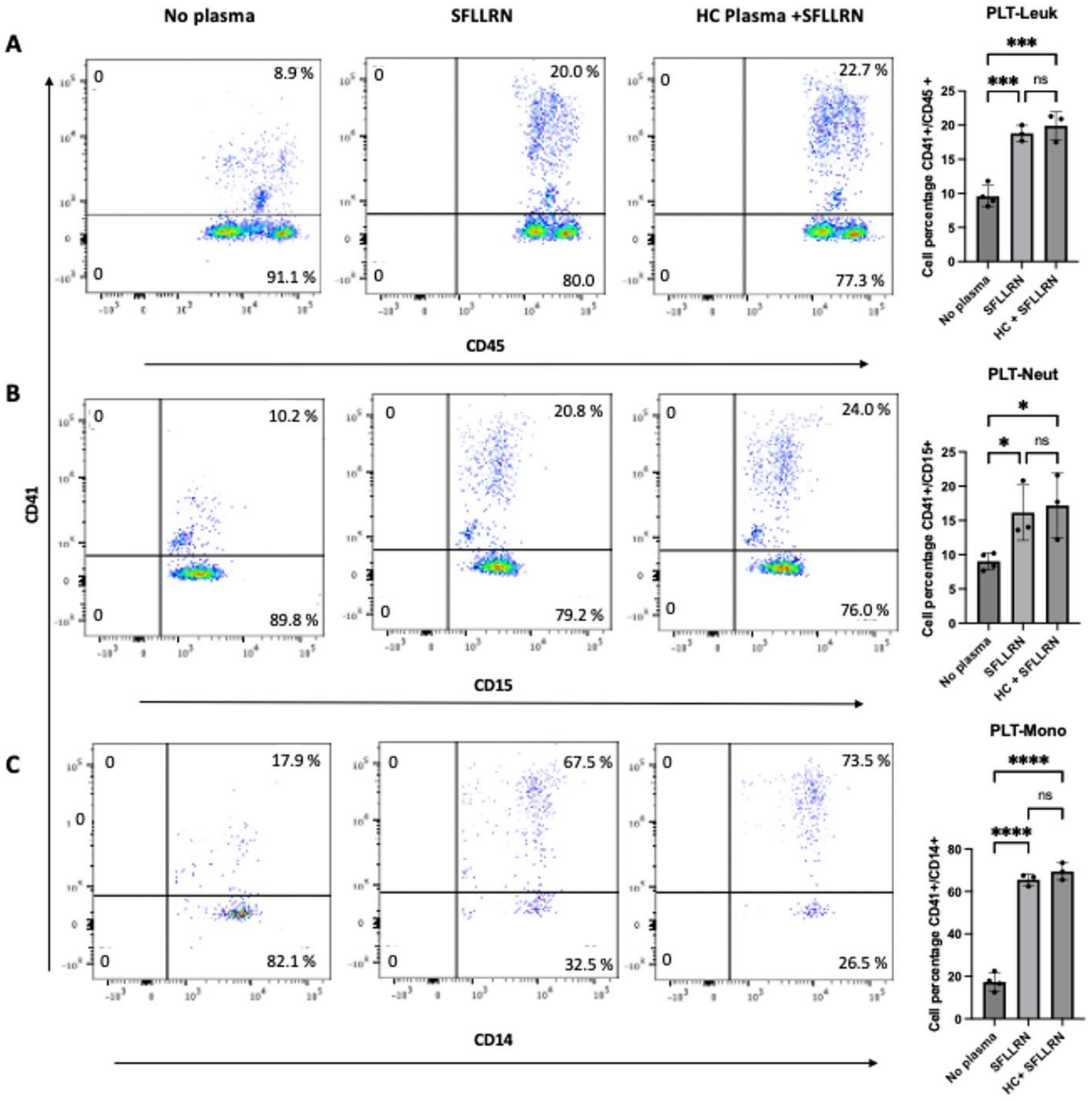


**Supplementary Figure 7-Platelet-Leukocyte interactions are induced by PAR-1 agonist SFLLRN, but no further increase is seen with healthy donor plasma.** Healthy donor (HD) whole blood was incubated with either vehicle or 5 µM SFLLRN or HD plasma and 5 µM SFLLRN. PLT-Leukocyte interactions (CD41/CD45 double positive) [**A**], PLT-Neutrophil interactions (CD41/CD15 double positive) [**B**], and PLT-Monocyte interactions (CD41/CD14 double positive) [**C**] were assessed by flow

cytometry. Representative dot plots are shown. *P<0.05, *** P<0.001, ****P<0.0001, ns= non-significant. PLT, platelet.

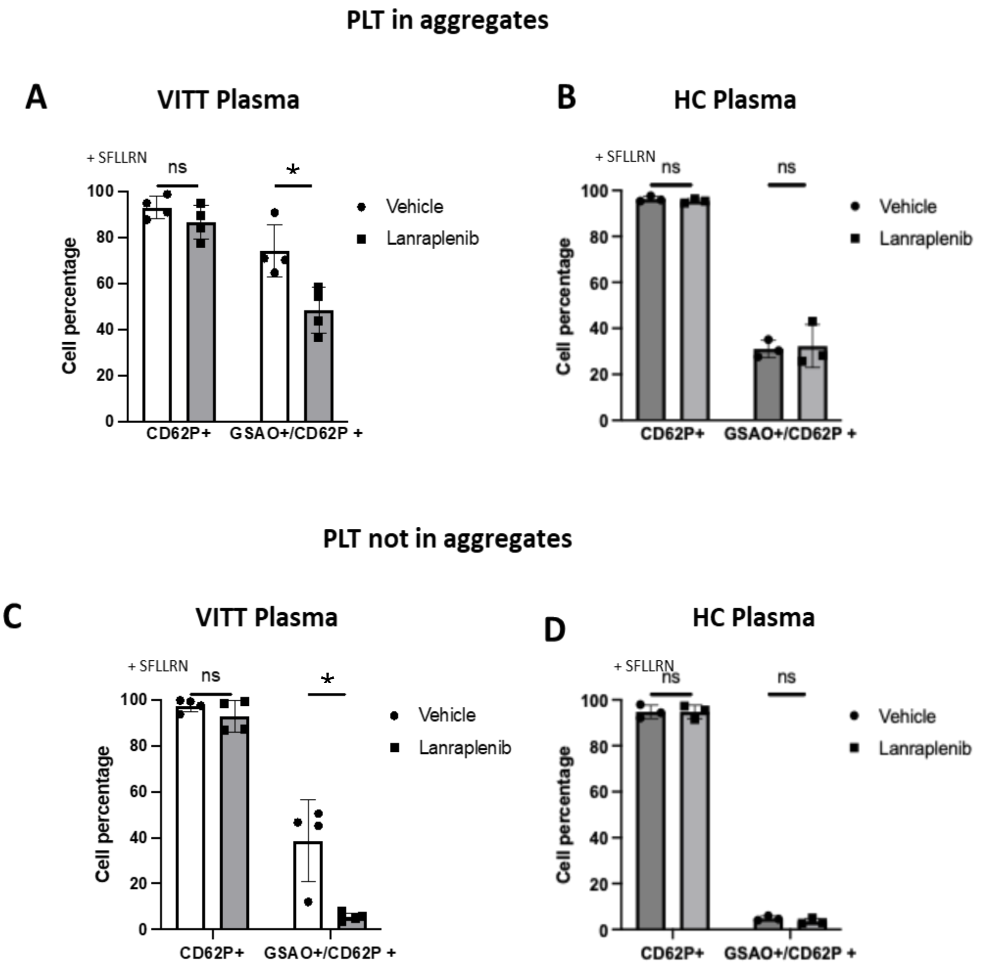


**Supplementary Figure 8- SYK inhibition reduced VITT-induced procoagulant PLT formation in both PLT-leukocyte aggregates and PLTs not in aggregates, but had no effect with healthy control plasma treated samples.** Samples were gated on CD41/CD45 double positive (PLT-leukocyte aggregates) [**A+B**]; or CD41 positive/CD45 negative events (PLTs not in aggregates) [**C+D**]. CD62p (P-selectin) expressing events (CD62p positive/GSAO-AF647 negative) and (CD62p /GSAO-AF647 double positive) were compared with procoagulant PLT events (CD62p/GSAO

double positive) in healthy donor whole blood preincubated with Lanraplenib (5 µM) or vehicle  and stimulated with 5 µM SFLLRN and VITT (**A+C**) or HC plasma (**B+D**). Note that CD62p positive events are the combination of CD62p single positive and CD62p/GSAO double positive events. Combined data reported in bar graphs showing the distribution of the values from N=4 (VITT) or N=3 (HC) individual plasma samples. Paired t-tests are shown. ns: non-significant. HC, healthy control; PLT, platelet.

# Supplementary Figure 9A

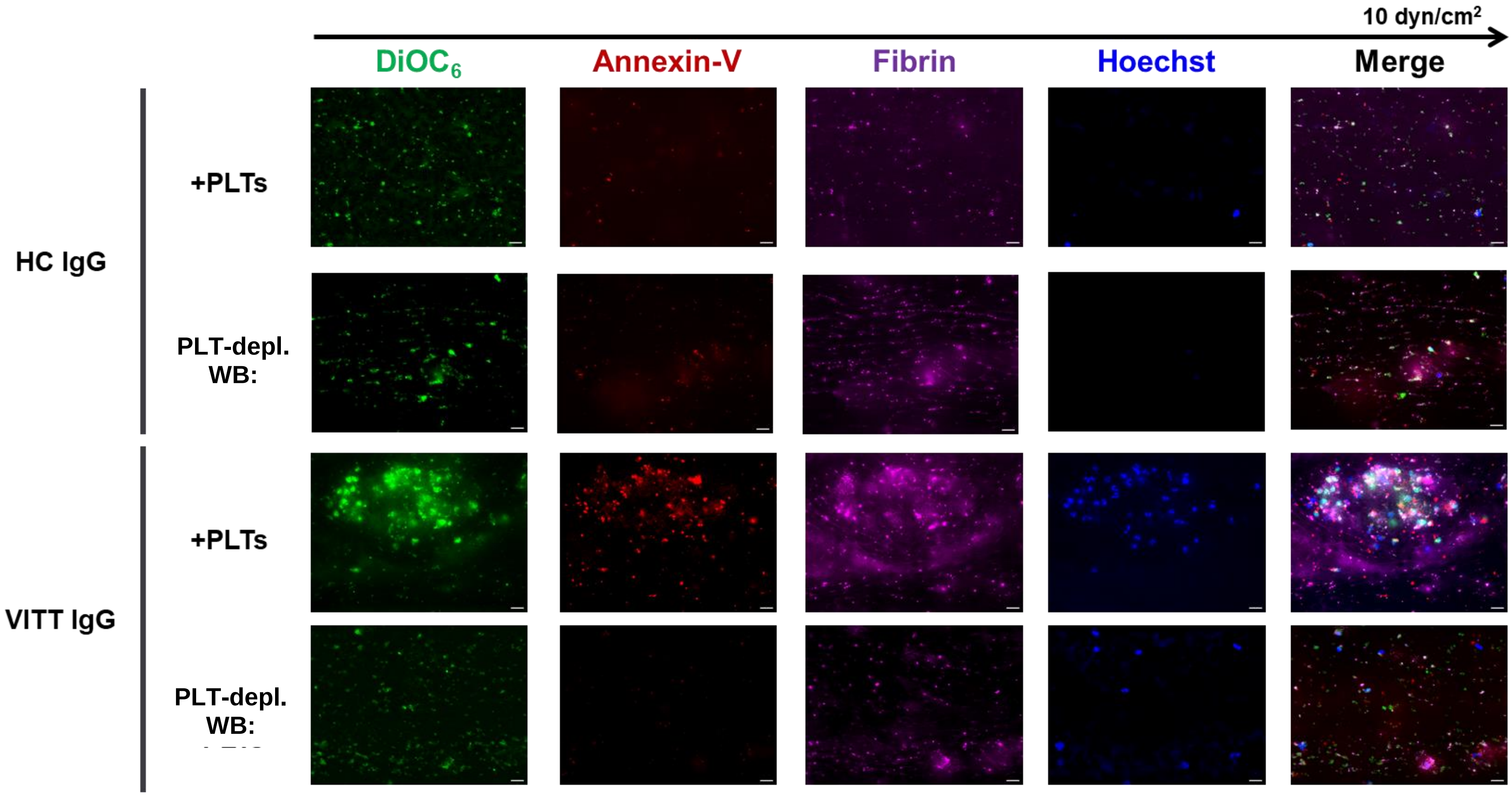

# Supplementary Figure 9B

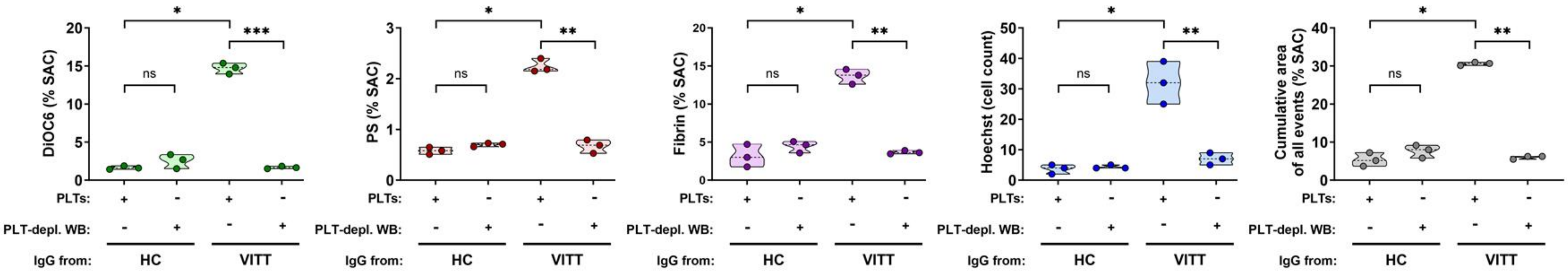


**Supplementary Figure 9-VITT antibody-induced procoagulant platelets are the main mediator of VITT antibody-mediated thrombus** Healthy control (HC, [N=3]) or VITT patient IgG (N=1) were incubated with platelets (indicated as +PLTs) or platelet-depleted whole blood (indicated as +PLT-depl. WB) in the presence of exogenous PF4 (10 µg/mL) prior to reconstitution with autologous WB (+PLTs) or reconstitution with autologous unstimulated PLTs (+PLT-depl. WB) and perfusion through microfluidic channels at a venous shear rate of $250s^{-1}$ (10 dyn/$cm^2$) for 25 minutes. [**A**] After perfusion, images were acquired at x40 magnification. Scale bar 20 µm. [**B**] Violin plots showing the percentage of total surface area coverage (% SAC) by DiOC6, phosphatidylserine (PS), Fibrin (-ogen), count of Hoechst-positive labeled cells and cumulative total % SAC with DiOC6, PS and Fibrin(-ogen) labeled thrombus captured in the microfluidic channel. The panels show results of the individual experiments using different donors. ns: non-significant. *P<0.05, **P<0.01 and ***P<0.001. VITT, vaccine-induced immune thrombotic thrombocytopenia; PF4, platelet factor 4; %SAC, surface area covered; IgG, immunoglobulin G; PF4, platelet factor 4.

# Supplementary Figure 10

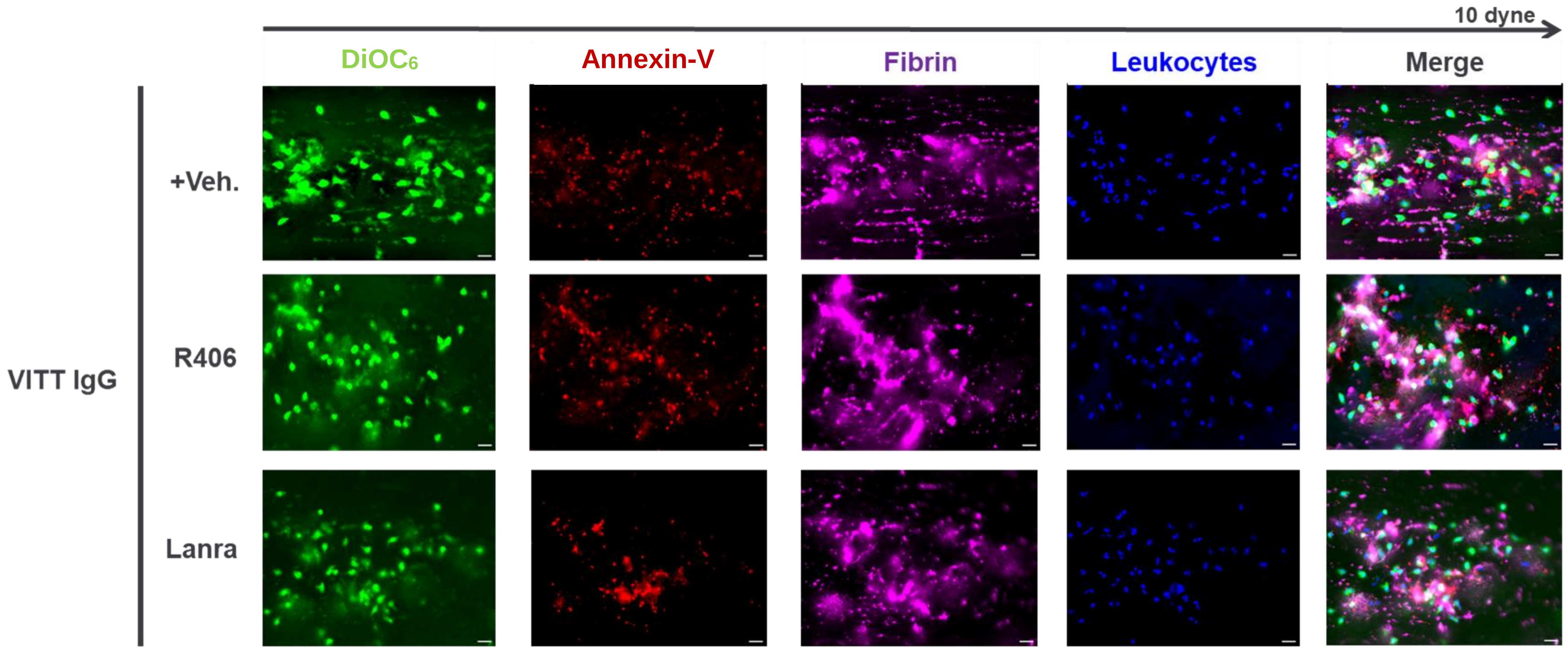


**Supplementary Figure 10-Pretreatment of leukocytes with SYK inhibitors does not prevent from VITT antibody-induced procoagulant platelet-mediated thrombus formation.** Platelet depleted whole blood from healthy individuals was pretreated with vehicle, R406 (5 μM) or Lanraplenib (5 μM) for 30 min at RT prior to reconstitution with VITT Ab-induced procoagulant platelets and perfusion through microfluidic channels at a venous shear rate of 250$s^{-1}$ (10 dyn/$cm^2$) for 25 minutes. After perfusion, images were acquired at x40 magnification. Scale bar 20 μm.

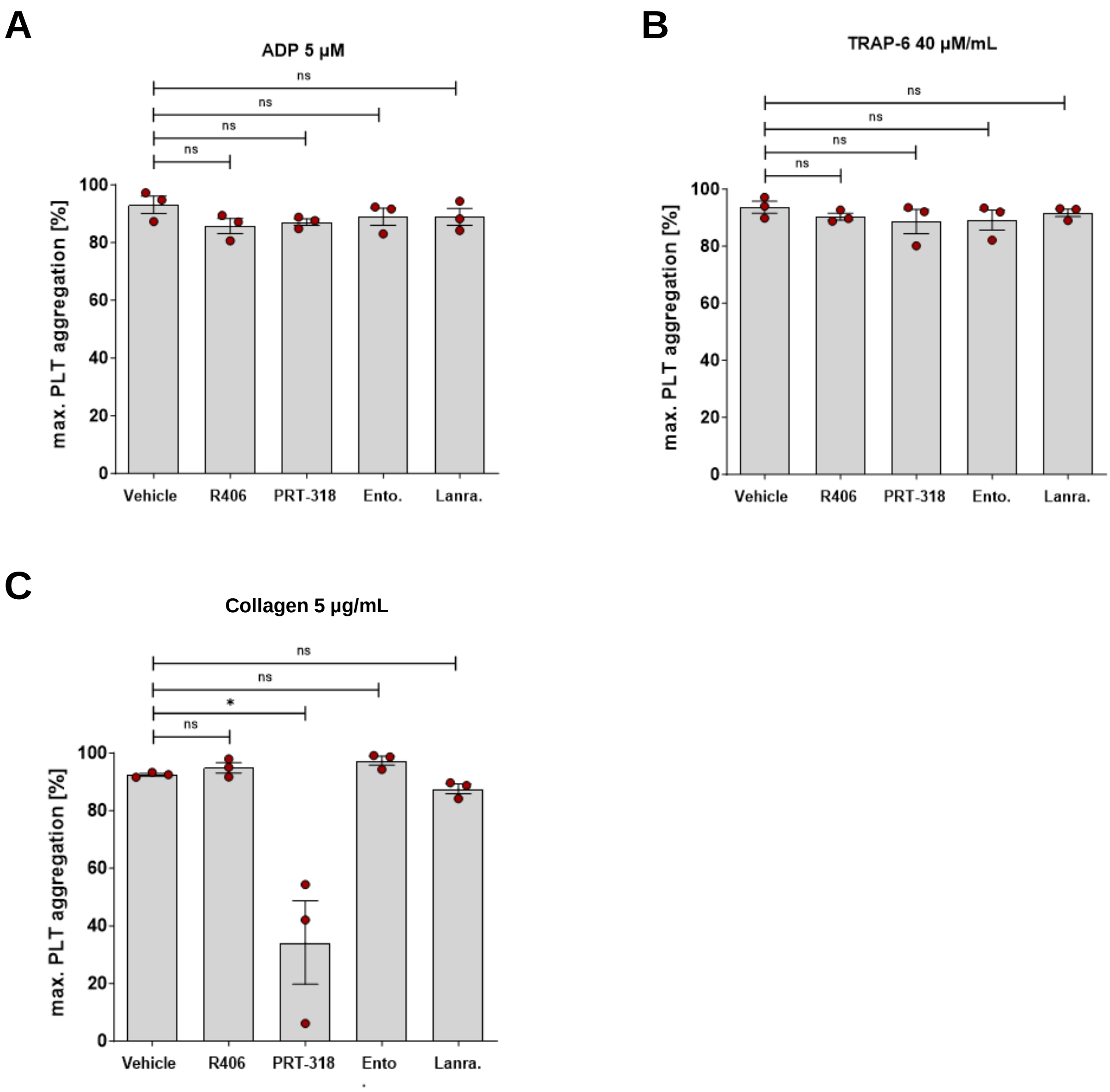


**Supplementary Figure 11-Impact of platelet SYK inhibition on platelet aggregation.** PRP from (N=3) healthy individuals was adjusted with autologous PPP to 300x$10^3$/µL and preincubated with vehicle (DMSO), or SYK inhibitors R406 (5 µM), PRT-060318 (1 µM), Entospletinib and Lanraplenib (both 5 µM) for 30 min at RT prior to stimulation with (**A**) ADP (5 µM), (**B**) TRAP-6 (40 µM) and (**C**) collagen (5 µg/mL) in

light transmission aggregometry. ns: non-significant. *P<0.05. PPP, platelet-poor plasma; SYK, spleen tyrosine kinase.

| Class | Inhibitors | VITT (n=10) |
|---|---|---|
| SYK inhibitor | R406 | N/A |
| SYK inhibitor | PRT060318 | 0.563 µM |
| SYK inhibitor | Entospletinib | 1.031 µM |
| SYK inhibitor | Lanraplenib | 0.941 µM |

**Supplementary Table 1**. Half-maximal inhibitory concentration of SYK inhibitors. Non-linear regression with variable slope and four parameters was used for IC50 determination. SYK, spleen tyrosine kinase; VITT, vaccine-induced immune thrombotic thrombocytopenia.


1. Uzun G, Althaus K, Hammer S, et al. Diagnostic Performance of a Particle Gel Immunoassay in Vaccine-Induced Immune Thrombotic Thrombocytopenia. *Hamostaseologie*. 2023;43(1):22-27.

2. Althaus K, Moller P, Uzun G, et al. Antibody-mediated procoagulant platelets in SARS-CoV-2-vaccination associated immune thrombotic thrombocytopenia. *Haematologica*. 2021;106(8):2170-2179.

3. Lee CSM, Liang HPH, Connor DE, et al. A novel flow cytometry procoagulant assay for diagnosis of vaccine-induced immune thrombotic thrombocytopenia. *Blood Adv*. 2022;6(11):3494-3506.

4. Lee CSM, Powell MC, Chen VM. Whole Blood Procoagulant Platelet Flow Cytometry Protocol for Heparin-Induced Thrombocytopenia (HIT) and Vaccine-Induced Immune Thrombotic Thrombocytopenia (VITT) Testing. *Methods Mol Biol*. 2023;2663:441-461.

5. Zlamal J, Singh A, Weich K, et al. Platelet phosphatidylserine is the critical mediator of thrombosis in heparin-induced thrombocytopenia. *Haematologica*. 2023;108(10):2690-2702.

6. Zlamal J, Althaus K, Jaffal H, et al. Upregulation of cAMP prevents antibody-mediated thrombus formation in COVID-19. *Blood Adv*. 2022;6(1):248-258.

7. Schindelin J, Arganda-Carreras I, Frise E, et al. Fiji: an open-source platform for biological-image analysis. *Nat Methods*. 2012;9(7):676-682.

8. Singh A, Toma F, Uzun G, et al. The interaction between anti-PF4 antibodies and anticoagulants in vaccine-induced thrombotic thrombocytopenia. *Blood*. 2022;139(23):3430-3438.

9. Agbani EO, Williams CM, Hers I, Poole AW. Membrane Ballooning in Aggregated Platelets is Synchronised and Mediates a Surge in Microvesiculation. *Sci Rep*. 2017;7(1):2770.

10. Josefsson EC, Ramstrom S, Thaler J, Lordkipanidze M, group Cs. Consensus report on markers to distinguish procoagulant platelets from apoptotic platelets: communication from the Scientific and Standardization Committee of the ISTH. *J Thromb Haemost*. 2023;21(8):2291-2299.
